# Adenylosuccinate lyase is oncogenic in colorectal cancer by causing mitochondrial dysfunction and independent activation of NRF2 and mTOR-MYC-axis

**DOI:** 10.7150/thno.50051

**Published:** 2021-02-15

**Authors:** Stephanie Taha-Mehlitz, Gaia Bianco, Mairene Coto-Llerena, Venkatesh Kancherla, Glenn R. Bantug, John Gallon, Caner Ercan, Federica Panebianco, Serenella Eppenberger-Castori, Marco von Strauss, Sebastian Staubli, Martin Bolli, Ralph Peterli, Matthias S. Matter, Luigi M. Terracciano, Markus von Flüe, Charlotte K.Y. Ng, Savas D Soysal, Otto Kollmar, Salvatore Piscuoglio

**Affiliations:** 1 Visceral Surgery and Precision Medicine Research Laboratory, Department of Biomedicine, University of Basel, Basel, Switzerland.; 2 Clarunis, Department of Visceral Surgery, University Center for Gastrointestinal and Liver Diseases, St. Clara Hospital and University Hospital Basel, Switzerland.; 3 Institute of Medical Genetics and Pathology, University Hospital Basel, Basel, Switzerland.; 4 Department of Biomedicine, Immunobiology, University of Basel, Basel, Switzerland.; 5 Department of Pathology, Humanitas Clinical and Research Center, IRCCS, Milan, Italy.; 6 Humanitas University, Department of Biomedical Sciences, Milan, Italy.; 7 Department for BioMedical Research, University of Bern, Bern, Switzerland.

**Keywords:** colorectal cancer, ADSL, mitochondria, fumarate, mTOR-MYC-axis

## Abstract

**Rationale:** Adenylosuccinate lyase (ADSL) is an essential enzyme for *de novo* purine biosynthesis. Here we sought to investigate the putative role of ADSL in colorectal carcinoma (CRC) carcinogenesis and response to antimetabolites.

**Methods:** ADSL expression levels were assessed by immunohistochemistry or retrieved from The Cancer Genome Atlas (TCGA) dataset. The effects of ADSL silencing or overexpression were evaluated on CRC cell proliferation, cell migration and cell-cycle. *In vivo* tumor growth was assessed by the chicken chorioallantoic membrane (CAM). Transfected cell lines or patient-derived organoids (PDO) were treated with 5-fluorouracil (5-FU) and 6-mercaptopurine (6-MP) and drug response was correlated with ADSL expression levels. Metabolomic and transcriptomic profiling were performed to identify dysregulated pathways and ADSL downstream effectors. Mitochondrial respiration and glycolytic capacity were measured using Seahorse; mitochondrial membrane potential and the accumulation of ROS were measured by FACS using MitoTracker Red and MitoSOX staining, respectively. Activation of canonical pathways was assessed by immunohistochemistry and immunoblotting.

**Results:** ADSL expression is significantly increased in CRC tumors compared to non-tumor tissue. ADSL-high CRCs show upregulation of genes involved in DNA synthesis, DNA repair and cell cycle. Accordingly, ADSL overexpression accelerated progression through the cell cycle and significantly increased proliferation and migration in CRC cell lines. Additionally, ADSL expression increased tumor growth* in vivo* and sensitized CRCs to 6-MP* in vitro*, *ex vivo* (PDOs) and *in vivo* (CAM model). ADSL exerts its oncogenic function by affecting mitochondrial function via alteration of the TCA cycle and impairment of mitochondrial respiration. The KEAP1-NRF2 and mTORC1-cMyc axis are independently activated upon ADSL overexpression and may favor the survival and proliferation of ROS-accumulating cells, favoring DNA damage and tumorigenesis.

**Conclusions:** Our results suggest that ADSL is a novel oncogene in CRC, modulating mitochondrial function, metabolism and oxidative stress, thus promoting cell cycle progression, proliferation and migration. Our results also suggest that ADSL is a predictive biomarker of response to 6-mercaptopurine in the pre-clinical setting.

## Introduction

Glucose and nucleotide metabolism are altered in cancer to promote cell reprogramming and survival under stress conditions 1. *De novo* purine nucleotide synthesis, in particular, has been shown to play a critical role in cancer cell growth under nutrient-limited conditions, a common feature of the tumor microenvironment 2,3. Adenylosuccinate lyase (ADSL) is an essential enzyme for *de novo* purine biosynthesis and the purine nucleotide cycle 4,5, where it plays a key role in the regulation of adenosine monophosphate levels in the cells. Given its crucial role in both cellular replication and metabolism, it is not surprising that ADSL has been found dysregulated in several malignancies, including breast 6 and endometrial 6,7 cancers as well as glioma 8. In particular, *ADSL* expression has been shown to be significantly upregulated and to be oncogenic in triple-negative breast cancer by enhancing tumor growth and invasiveness, at least partially through the regulation of the long non-coding RNA *MIR22HG* and, indirectly, *cMYC* and *cMYC* target genes expression 6. Similarly, *ADSL* has been shown to enhance the aggressiveness of endometrial cancer cells by increasing cell proliferation and migration, as well as invasive potential via the regulation of the natural killer cells lectin-like receptor C3 (KLRC3) expression by fumarate 7.

The use of antimetabolites is a common strategy in the treatment of cancer 9,10. Antimetabolites are small molecules that resemble nucleotide metabolites and act by inhibiting the activity of enzymes involved in DNA synthesis 10. Fluorouracil (5-FU), a pyrimidine analogue 9,11, is a notable example and is the most commonly used constituent of chemotherapy combination regimens to treat colorectal cancers (CRC) 9,12. However, almost half of CRC patients develop resistance to 5-FU-based chemotherapy 13,14. A previous study found that *ADSL* activity was increased in pre-neoplastic colonic lesions 15, but a comprehensive assessment of ADSL in CRC has not been performed. In this study, we sought to define the putative role of ADSL in CRC carcinogenesis and response to 5-FU using a combination of both *in vitro, ex vivo,* and *in vivo* models. Furthermore, given the central role of ADSL in *de novo* purine synthesis, we evaluated ADSL expression as a predictive biomarker of response to the purine analogue 6-mercaptopurine (6-MP).

## Methods

### Patients and specimen characteristics

One hundred primary non-consecutive CRCs treated at the University Hospital Basel between the years 2006 and 2012 were included in this study. A tissue microarray (TMA) of these 100 tumors was constructed. For thirty cases non-malignant adjacent mucosa was selected from the same donor block (**Supplementary Methods**). Forty-three primary CRCs and forty-five metastases, including matched primary tumors and metastases of thirty-seven patients, treated at the University Hospital Basel between the years 1995 and 2015 were also used to construct a second TMA. Both studies were performed in accordance with the Declaration of Helsinki and approved by the ethics committee (Ethics Committee of Basel, EKBB 361/12). Data were collected retrospectively in a non-stratified and non-matched manner including patient age, gender, location, stage, grade, vascular invasion, and clinical outcome.

### Analysis of The Cancer Genome Atlas (TCGA) dataset

Gene-level expression data for the TCGA CRC cohort 16 with 622 tumors and 51 normal tissues were obtained from Genomics Data Commons (**Supplementary Methods**). Tumor samples were classified as *ADSL*-overexpressing (n = 218) and non-*ADSL*-overexpressing (n = 404) based on the threshold of mean + 2 standard deviations of the normal tissues. Clinical information was obtained for 596 CRCs. Differential expression analysis was performed using the *edgeR* package 17. Gene set enrichment analysis was performed using the *fgsea* 18 package with genes ranked based on signed p-value according to the direction of the log-fold change (**Supplementary Methods**).

### Cell culture

CRC-derived cell lines (HT-29, SW480, Caco-2, and DLD-1) were authenticated by short tandem repeats and cultures were confirmed to be free of mycoplasma infection using the PCR-based Universal Mycoplasma Detection kit (**Supplementary Methods**). NCM460 normal colonic cell line was purchased from INCELL Corporation and cultured according to manufacturer's instructions.

### Transient gene knockdown and overexpression

Transient gene knockdown was conducted using ON-TARGET plus siRNA transfection. Gene overexpression was conducted using jetPRIME™ (#9154226, Polyplus-transfection, **Supplementary Methods**).

### Immunoblot

Total protein lysates containing protease and phosphatase inhibitors were treated with reducing agent, loading buffer, boiled and loaded into SDS-PAGE gels (4-12%). Proteins were transferred to nitrocellulose membranes, blocked for 1 h and incubated overnight at 4 °C with the following primary antibodies: anti-ADSL (1 : 750; A000525, Sigma), anti-B actin (1 : 5000; A5441; Sigma), anti-c-Myc (1 : 1000, MA1-980, ThermoFisher), anti-S6 (1 : 1000, 2217S, Cell Signaling), anti-phospho-S6 (Ser235/236) (1 : 2000, 4858S, Cell Signaling), anti 2-SC antibody (crb2005017d, Discovery Antibodies), anti-NRF2 (1 : 1000, D1C9, Cell Signaling), anti-KEAP1 (1 : 1000, D6B12, Cell Signaling) and anti-TOM20 (1 : 1000, D8T4N, Cell Signaling). Membranes were subsequently incubated with secondary goat anti-mouse or anti-rabbit antibodies for 1 h at room temperature. Band intensity was quantified using ImageJ software and the ratio of proteins of interest/loading control was normalized to their control counterparts (**Supplementary Methods**).

### Cell proliferation assay

Cell proliferation was assessed using CellTiter-Glo^TM^ Luminescent Cell Viability Assay (#G7570, Promega, **Supplementary Methods**). For drug treatment, transfected cells were re-plated in 96-well plates, and after 4 h, 5-Fluorouracil (5-FU) (#S1209, Selleckchem), 6-MP (#S1305, Selleckchem) or equal concentration of vehicle (DMSO) was added. After 48 h of treatment, cell proliferation was assayed using CellTiter-Glo^TM^. Results were normalized to the vehicle.

### Migration assay

Cell migration was assayed using the xCELLigence Real-Time Cell Analyzer (RTCA) DP (ACEA Biosciences, **Supplementary Methods**). All experiments were performed in triplicate. Results are shown as mean±SD. Statistical significance was assessed by multiple t-test.

### Cell cycle analysis by flow cytometry

Cells were transfected with control or ADSL encoding plasmid. 24 h post transfection transfection medium was replaced by either complete medium or glucose-depleted medium and the cells were left to grow for an additional 48 h. Cells were stained with DAPI (10236276001, Sigma-Aldrich) and analyzed by flow cytometry using the BD FACS Canto II cytometer (BD Biosciences). Data were analyzed using the FlowJo software version 10.5.3 (https://www.flowjo.com,** Supplementary Methods**).

### Generation of CRC patient-derived organoids (PDOs)

CRC-patient liver metastases were obtained from the University Hospital Basel following patient consent and ethical approval (Ethics Committee of Basel, EKBB, number EKBB 2019-02118). Surgically resected tissues were transported to the laboratory in ice-cold MACS buffer (cat #130-100-008, Miltenyi) and were processed as previously described 19,20 (**Supplementary Methods**). The medium was supplemented with growth factors as previously described 21.

### Drug Treatment in PDOs

Tumor organoids were plated as single cells in a 96-well plate at a density of 1 × 10^4^ cells in 10 μL Matrigel droplets. Before treatment, cells were allowed to recover and form organoids for 2 to 3 days. At day 3, 6-MP at a final concentration of 2.5 µM or 5-FU at concentrations of 1.25, 2.5 and 5 µM or in combination was added to the medium, and cell viability was assessed after 5 day using CellTiter-Glo 3D reagent (#G9682, Promega). Luminescence was measured on Varioskan Microplate Reader (ThermoFisher Scientific). Results were normalized to DMSO control. All experiments were performed twice in quadruplicate.

### Chorioallantoic membrane (CAM) assay

Fertilized chicken eggs were obtained at day 1 of gestation and incubated at 37 °C with 60% humidity for 9 days. At this time, an artificial air sac was formed, and the CAM was accessed as previously described 22 (**Supplementary Methods**). Caco-2 cells growing in tissue culture were inoculated on CAMs at 2 × 10^6^ cells per CAM, on three to four CAMs each. Embryos were maintained at 37 °C for 4 days after which tumors at the site of inoculation were excised using surgical forceps. Images of each tumor were acquired using a Canon EOS 1100D digital camera. Tumor size measurements were performed by averaging the volume (height*width*width) of each tumor using ImageJ, as previously described 23.

### Immunohistochemistry (IHC)

Colonic cell lines were harvested by scraping with cold PBS and fixed in 10% Paraformaldehyde (PFA) for 30 min at room temperature. Similarly, immediately after excision, CAM assay-explanted tumors were fixed in 10% PFA for 2 days at room temperature. PFA-fixed and paraffin-embedded cell pellets and tumors were cut as 4 μm-thick sections. Sections were stained with hematoxylin and eosin (H&E) according to standard protocols.

Immunohistochemical staining was performed on a Benchmark immunohistochemistry staining system (Bond, Leica) with BOND polymer refine detection solution for DAB, using anti-ADSL (1 : 100, A000525, Sigma HP), anti-cleaved caspase 3 (1 : 200, 9661S, Cell signaling), anti-c-Myc (1 : 200, MA1-980, ThermoFisher) and anti-phospho-S6 (Ser235/236) (1 : 400, 4858S, Cell signaling) primary antibodies as substrate. Images were acquired using an Olympus BX46 microscope.

For the analysis of the CRC TMA, ADSL immunoreactivity was scored semi-quantitatively by multiplying the proportion of ADSL positive cells (%) and the staining intensity (0 = none; 1 = weak; 2 = intermediate; 3 = strong). The median value of the score in the tumor tissues (score median = 40) was used as cut-off and samples containing a score lower than the median were defined as ADSL-low (ADSL score < 40) while samples containing a score higher than the median were defined as ADSL-high (ADSL score > 40). For all tumors, the tumor-stroma content was calculated as described previously 24 (**Supplementary Methods**).

For the quantification of cleaved caspase 3 (Cl.Casp3) staining performed on the tumors extracted from the CAMs, Cl.Casp3 positive cells were counted in a single high power field (HPF) with the highest density of positive cells, at 400x magnification.

### RNA extraction and quantitative PCR

RNA extraction from snap-frozen tissues was performed using TRIzol Reagent (Invitrogen) according to manufacturer's guidelines (Supplementary Methods). cDNA was synthesized from 400 ng of total RNA. Quantitative RT-PCR analysis was performed using SYBR Green. Expression of GAPDH was used to normalize. mRNA fold expression change was calculated by the 2-ΔΔCT method as previously described 25. All samples were analyzed in triplicate.

### Metabolite extraction and multiple pathway targeted analysis (LC-MS Analysis)

Cell lysates were extracted and homogenized as previously described 26,27 (**Supplementary Methods**). Dried sample extracts were re-suspended in MeOH:H_2_O (4 : 1, v/v) prior to LC-MS/MS analysis according to the total protein content. Extracted samples were analyzed by hydrophilic interaction liquid chromatography coupled to tandem mass spectrometry (HILIC - MS/MS) in both positive and negative ionization modes as previously described 28 (**Supplementary Methods**).

### Analysis of metabolomic data

Comparative analysis of metabolic profiles between ADSL overexpressing and control cells was carried out using MetaboDiff 29. Data were normalized using variance stabilizing normalization before comparing mean metabolite levels using the student's t test. P values were adjusted using the Benjamini Hochberg procedure. Metabolic correlation modules were defined using the weighted gene coexpression network analysis methodology 30, and identified modules were named according to the most abundant pathway annotation in the module. The metabolic correlation modules were then correlated with ADSL overexpression to identify modules significantly associated with the ADSL overexpressing phenotype.

### RNA sequencing and pathway analysis

Biological replicates were generated for all the samples analyzed. Total RNA was extracted from cells at 75% confluence using TRIZOL according to the manufacturer's guidelines. RNA was treated and library generated as described in **Supplementary material and methods**. Differential expression analysis was performed using the DESeq2 Wald test. Gene set enrichment analysis was performed using the *fgsea* R package 18 and the Hallmark gene set from the Molecular Signatures Database 31, using the ranked t statistics from the DESeq2 Wald test. Pathways with false discovery rate (FDR) < 0.05 were considered to be significant. Results were visualized using ggplot2 32.

### MitoTracker and MitoSOX staining

For the analysis of changes in mitochondrial membrane potential, 48 h post-transfection *ADSL* overexpressing and control Caco-2 cells were stained with Mitotracker Red (MTR, M22425, Thermo Fisher). Briefly, 5 × 10^5^ cells were resuspended in 100 μL of pre-warmed (37 °C) FACS buffer (0.1% BSA, dilute in PBS) and placed in a 96-well U plate. Then, 100 μL of MTR (100 nM) were added to the wells and cells were incubated 20 min at 37 °C. The reaction was stopped by addition of cold DMEM-10% FBS and incubating for 5 min at 4 °C. Cells were washed twice with DMEM-10% FBS and resuspend in 100 μL of FACS buffer.

For analysis of mitochondrial production of ROS, 48 h post-transfection control and *ADSL* overexpressing Caco-2 cells were stained with Mitosox red (M36008,Thermo Fisher). Briefly, 1 × 10^5^ cells were resuspended in 100 μL of pre-warmed HBSS (Ca^2+^ and Mg^2+^) and placed in a 96-well U plate. 100 μL of Mitosox red (20 μM) diluted in prewarmed HBSS was added to each well. Cells were incubated 20 min at 37 °C. After incubation, cells were washed twice with FACS buffer and resuspended in 100 μL of FACS buffer.

All samples were analyzed using the CytoFLEX cytometer (Beckman). Information about the percentage of positive cells as well as Mean Fluorescence Intensity were recorded for all the samples and analyzed by FlowJo software version 10.5.3.

### OCR and ECAR measurements

For analysis of the Oxygen consumption rate (OCR) (in pmol/min) and extracellular acidification rate (ECAR) (in mpH/min), the Seahorse XF-24 metabolic extracellular flux analyzer was used (Seahorse Bioscience). Caco-2 and *NCM40D* were plated onto Seahorse cell plates (8 × 10^4^ cells per well) in serum-free unbuffered DMEM medium (Sigma-Aldrich). Perturbation profiling of the use of metabolic pathways was achieved by the addition of oligomycin (1 μM), Carbonyl cyanide-4-(trifluoromethoxy)phenylhydrazone (**FCCP**) (0.5 μM) and rotenone (1.3 μM) plus antimycin A (20 μM) (all from Sigma-Aldrich). Experiments with the Seahorse system were done with the following assay conditions: 2 min mixture; 2 min wait; and 4-5 min measurement. Metabolic parameters were then calculated as previously described 33.

### Fumarate measurement

Fumarate concentration in Caco-2 and NCM460 cells was measured using the Fumarate Assay Kit (Abcam, 102516) following the manufacturer's instructions. Briefly, cells were plated in 96-well plates 8 h after transfection and supernatant was collected 24, 48, 72 and 96 h post-transfection. The amount of fumarate was quantified relative to fumarate standard curve.

### Fumarate rescue experiment

Caco-2 cells were re-plated at a density of 5 × 10^3^ cells/well in a 96-well plate 8 h post transfection. Dimethyl fumarate (Selleckchem, #S2586) or vehicle (DMSO) were added to cells 18 h before measuring the cell index for the respective time points to a final concentration of 50 µM. Proliferation was measured using the CellTiter-Glo^TM^ Luminescent Cell Viability Assay (Promega, #G7570) at 24, 48, 72 and 96 h after re-plating. Results were normalized to 4 h.

### Statistical analysis

Statistical analyses were performed using Prism software v6.0 (GraphPad Software) and R. For *in vitro*, *in vivo* and *ex vivo* studies, statistical significance was determined by the two-tailed unpaired Student's t-test. The differences were considered statistically significant at p < 0.05. All experiments were performed at least twice. The statistical parameters (e.g., the exact value of n, p values) have been noted in the figures and figure legends. Bar plots with error bars show mean ± SD. Statistical comparisons for categorical variables were performed using χ^2^ or Fisher's exact tests where appropriate. Statistical comparisons for numeric and ordinal variables were performed using t-test, Mann-Whitney U test, paired Wilcoxon test or Cochran-Armitage test for trend where appropriate. Ordinal regression analysis was performed using cumulative link models using the 'ordinal' R package. Survival analysis was performed using the Kaplan-Meier method and log-rank test. For the TCGA cohort, stratification of ADSL expression for overall survival analysis was performed using the 'maxstat' 34 R package.

## Results

### *ADSL* is overexpressed in CRCs but its expression does not increase with disease stage

**Figure 1:**
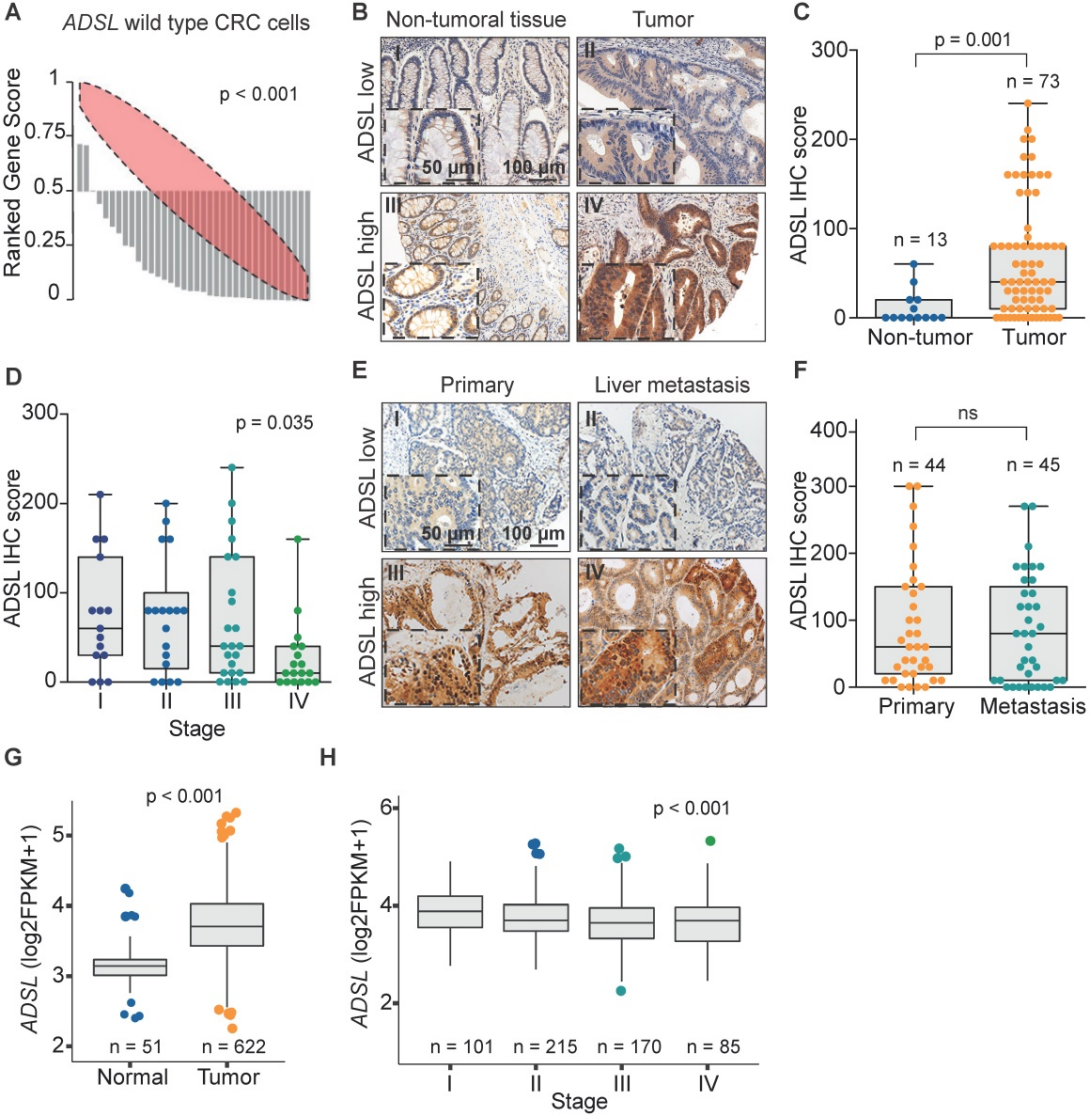
***ADSL* is overexpressed in CRC.** (**A**) Rank profile of cell viability upon knockdown of the *ADSL* gene in CRC cell lines with no genetic alteration in the *ADSL* gene. Each bar in the waterfall plot represents one cell line. The value of each bar represents the rank of viability for the *ADSL* gene among all genes knocked down for a given cell line in the project DRIVE. Thus ranks close to zero represent reduced viability while ranks close to one indicate cell growth upon knockdown of *ADSL*. The red ellipse in the rank profile represents a no-change (random) viability band. (**B**) Representative micrographs showing ADSL staining in CRC and non-tumoral adjacent tissue: (I) non-tumoral tissue with low ADSL staining score; (II) tumor tissue with low ADSL staining score*;* (III) non-tumoral tissue with high ADSL staining score and (IV) tumor tissue with high ADSL staining score. Semi-quantitative scoring of ADSL staining in (**C**) CRCs and non-tumoral adjacent tissues and (**D**) CRCs stratified by disease stage. (**E**) Representative micrographs showing ADSL staining in CRC primary and metastasis tissue: (I) primary tumor with low ADSL staining score; (II) metastasis tissue with low ADSL staining score*;* (III) primary tumor with high ADSL staining score and (IV) metastasis tissue with high ADSL staining score. (**F**) Semi-quantitative scoring of ADSL staining in CRC primary and metastasis tissue. (**G**) *ADSL* transcript expression in CRCs and normal tissues in the TCGA cohort 36. (**H**) *ADSL* transcript expression in CRCs in the TCGA cohort, stratified by disease stage. Statistical significance was assessed for (**C**,** F-G**) by unpaired student's t-tests and for (**D-H**) by ordinal regression. Scale bars 50-100 µm.

We recently analyzed the large-scale perturbation screen of the project DRIVE (Deep RNAi Interrogation of Viability Effects in cancer), in which 7,837 genes were targeted with a median of 20 shRNAs per gene in 398 cancer cell lines across a variety of malignancies 35. In this analysis, we identified the *ADSL* gene as one of the top putative oncogenes in CRC*.* Indeed, 33 out of 35 CRC cell lines profiled displayed significantly decreased cell viability upon *ADSL* knockdown (p < 0.001; **Figure 1A**). To confirm the putative oncogenic role of *ADSL* and its potential role as biomarker in CRC, we performed ADSL IHC on two TMAs, one containing 100 CRC samples and 30 non-tumoral adjacent tissues and the other containing 43 CRC primary samples and 45 metastatic tissues. In the first TMA, after excluding the samples with missing tissue core or poor staining, 73 tumor samples and 13 non-tumoral tissue cores were analyzed for ADSL protein expression. Immunoscore results showed that ADSL expression was significantly increased in tumor tissues compared to non-tumor tissue areas (p = 0.001; **Figure 1B-C**). Additionally, a regression analysis found that ADSL immune scores showed a downward trend with increasing disease stage (p = 0.035; **Figure 1D**). In the second TMA containing CRC primary and metastatic tissues, we observed no difference in ADSL expression levels between primary and all types of metastasis (liver, lung, small intestine, lymph node, abdominal wall, bone, bladder, cutaneous, ovarian; **Figure 1E-F** and**Figure S1A**) or between primary and paired liver metastasis (**Figure S1B**). Our results suggest that ADSL is overexpressed in CRC but its expression is not increased upon tumor progression.

We then asked whether ADSL expression was associated with any clinicopathological feature in our primary CRC patient cohort (**Table S1**). We found that ADSL-low tumors were associated with lymphatic and lymphovascular invasion, as well as with low tumor-stroma ratio (all p = 0.001; **Table S1**), all indicators of poor prognosis. No association with microsatellite instability, tumor grade (**Table S1**), or patient survival (**Figure S1C**) was observed.

To corroborate the results obtained with our in-house cohorts we evaluated the mRNA expression of *ADSL* in the 622 CRCs and 51 normal tissues in the TCGA cohort 36. In accordance with the IHC results, we found that *ADSL* expression was significantly higher in CRC samples compared to normal tissue (p < 0.001; **Figure 1G**). Additionally, similar to our TMA cohort of primary CRCs, ADSL transcript levels showed a downward trend with increasing disease stages (p < 0.001; **Figure 1H** and **Table S2**). However, the difference in *ADSL* expression levels between early- and advanced-stage patients was very modest (**Figure 1H**). We also found that ADSL levels were slightly higher in CRC tumors classified as CMS2 37 (p < 0.05 compared to other CMS subtypes, **Figure S2A**), a molecular subtype characterized by epithelial differentiation and strong upregulation of WNT and MYC downstream targets 37. No association between *ADSL* expression and patient overall survival was found (**Figure S2B**).

Taken together our data support the hypothesis that ADSL acts as a potential oncogene in CRC and suggest its possible implication in the early stage of colorectal carcinogenesis.

### *ADSL* has oncogenic-like properties in *in vitro* and* in vivo* models of CRC

**Figure 2:**
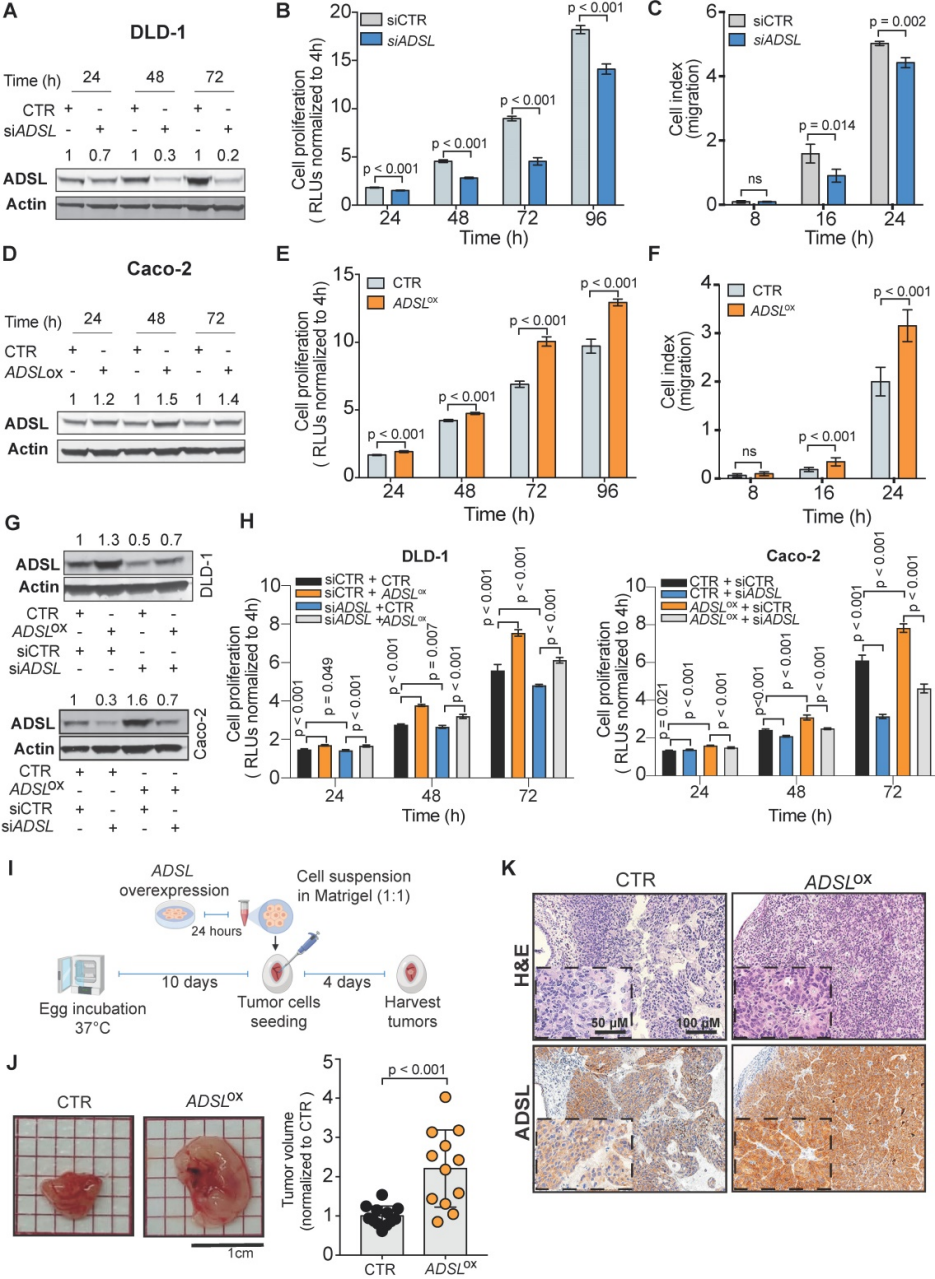
***ADSL* has oncogenic-like properties in *in-vitro* and* in-vivo* models of CRC** (**A**) Immunoblot showing ADSL expression in the DLD-1 cell line at 24, 48 and 72 h post siRNA transfection. (**B**) Proliferation and (**C**) migration capacity of DLD-1 *ADSL*-silenced cells compared to control cells. (**D**) Immunoblot showing ADSL expression in Caco-2 cells at 24, 48, and 72 h post vector transfection. (**E**) Proliferation and (**F**) migration capacity of Caco-2 *ADSL*-overexpressing cells compared to control cells. (**G**) Immunoblot showing ADSL expression in DLD-1 cells (upper panel) and Caco-2 cells (lower panel) 72 h post siRNA and/or vector transfection. (**H**) Rescue experiment showing proliferation capacity of DLD-1 (left) and Caco-2 (right) cells with siRNA and/or vector transfection compared to control cells. (**I**) Schematic illustration of the CAM assay. (**J**) Representative pictures and quantification of volume of Caco-2 control (CTR) and *ADSL*-overexpressed (*ADSL*ox) resected tumors 4 days post-implantation of cells in CAMs (n = 12 tumors from 2 independent experiments); mean ± SD. Values are normalized to the mean of CTR. (**K**) Representative pictures of Caco-2 tumors extracted 4 days post-implantation. Tissue sections were immunostained with ADSL. Scale bars 20-50 µm. For (**C**) and (**F**) 8, 16 and 24 correspond to 32, 40 and 48 h post transfection, respectively.

To validate the potential oncogenic role of *ADSL* we screened ADSL protein expression in CRC cell lines and selected the SW480 and DLD-1 cell lines, with high endogenous levels*,* for knock-down experiments, and the Caco-2 and HT-29 cells, with low endogenous levels, for overexpression (**Figure S3A-B**). We first sought to validate the results from the project DRIVE and assessed if *ADSL* knock-down would affect cell viability in CRC. By silencing *ADSL* using a siRNA approach, we achieved a reduction of ADSL protein expression by 70% and 80% 48 h post-transfection in DLD-1 and SW480 cells, respectively (**Figure 2A** and**Figure S3C**). In both cell lines, transient *ADSL* knock-down significantly decreased the proliferation rate compared to control cells (**Figure 2B** and**Figure S3D**). Similarly, both DLD-1 and SW480 *ADSL*-silenced cells showed a significant reduction in the migration potential compared to control cells (**Figure 2C** and**Figure S3E**). These data support our hypothesis that ADSL is a novel oncogene in CRC. However, the phenotype observed upon *ADSL* depletion may also imply that *ADSL* is an essential gene in colonic cells. To test this hypothesis, we silenced *ADSL* expression in the normal colonic cell line NCM460 (**Figure S3F**). Of note, ADSL levels are already lower in normal cells compared to colorectal tumor cells (**Figure S3A**). *ADSL* gene knock-down did not significantly impact the viability of NCM460 cells (**Figure S3G**), suggesting that while it confers a proliferative advantage to tumor cells, it does not have the same effect/impact in normal colonic cells.

To mimic the conditions observed in human CRC tissues and to validate the oncogenic role of ADSL, we assessed cell proliferation and migration potential upon forced overexpression of *ADSL* in Caco-2 and HT-29 cells. We achieved 35% and 25% increased ADSL protein expression 48 h post-transfection in Caco-2 and HT-29 cells, respectively (**Figure 2D** and**Figure S3H**). In support of our hypothesis, overexpression of *ADSL* in Caco-2 and HT-29 led to a significant increase in the proliferation rate compared to control cells (**Figure 2E** and**Figure S3I**). Similarly, the migration rate was significantly increased in *ADSL*-overexpressing cells compared to control cells (**Figure 2F** and**Figure S3J**).

To demonstrate the specificity of our results, we performed rescue experiments. Specifically, we restored ADSL expression levels in DLD-1 and Caco-2 cells where *ADSL* was silenced or overexpressed, respectively (**Figure 2G**). Restoring ADSL to levels similar to the endogenous expression rescued the phenotype, thus suggesting that the effects induced by *ADSL* modulation on cell proliferation were on-target (**Figure 2H**).

To further demonstrate the putative oncogenic role of ADSL in CRC, we modulated *ADSL* expression and xeno-transplanted *ADSL*-overexpressing or control CRC cells into the chicken chorioallantoic membrane (CAM) to assess tumor growth *in vivo*. Engraftment of tumor cells in the CAM has been successfully used as a fast and reproducible model of tumorigenesis 38,39. Briefly, twenty-four hours post-transfection, CRC cells were harvested, re-suspended in Matrigel and seeded into the CAMs (**Methods**). Four days later, the eggs were screened for tumor formation and tumors were harvested (**Figure 2I**). *ADSL*-overexpressing Caco-2 cells formed significantly larger tumors compared to control cells (**Figure 2J**). IHC analysis confirmed that the resected tumors were indeed of human origin and that ADSL overexpression could still be detected five days post-transfection (**Figure 2K** and **Figure S3K**).

Our *in vitro* and *in vivo* results provide evidence of the role of ADSL as an oncogene in colorectal carcinogenesis.

### Dysregulation of ADSL affects cell cycle progression and increases the DNA damage marker γH2AX

**Figure 3:**
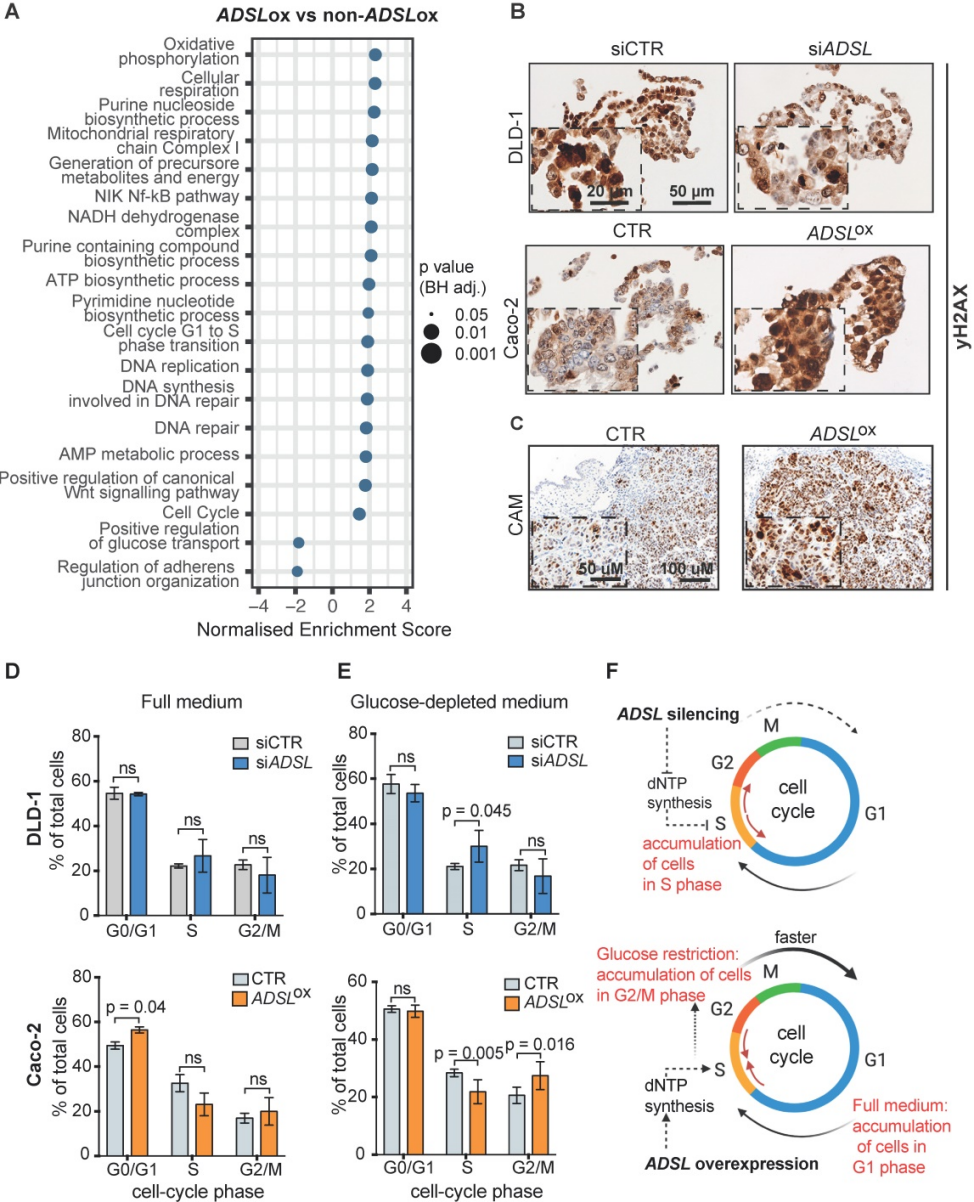
** Dysregulation of ADSL affects cell cycle and induces replication stress** (**A**) Normalized enrichment scores of significantly up-regulated pathways identified by gene set enrichment analysis (GSEA) in *ADSL*-overexpressing (*ADSL*ox) versus non-*ADSL*-overexpressing (non-*ADSL*ox) CRCs. NES = normalized enrichment score. (**B**) Representative pictures of control and *ADSL*-silenced DLD-1 cells and control *ADSL*-overexpressed Caco-2 cells immunostained with the DNA damage marker γH2AX. Scale bars 20-50 µm. (**C**) Representative pictures of Caco-2 tumors extracted 4 days post-implantation. Tissue sections were immunostained with γH2AX. Scale bars 50-100 µm. (**D-E**) Flow cytometry analysis of DAPI-stained DLD-1 (blue) and Caco-2 (orange) upon *ADSL* transient downregulation or upregulation in (**D**) complete medium and (**E**) glucose-deprived medium. (**F**) Schematic representation of *ADSL* silencing or *ADSL* overexpression impact on cell cycle in CRC cell lines. Data are mean ± SD (**D-E**) n ≥ 3 replicates. For all experiments, statistical significance was assessed by multiple t-tests.

To understand which signaling pathways might be affected as a result of *ADSL* overexpression we classified CRC patient samples from the TCGA cohort as *ADSL*-overexpressing and non-*ADSL*-overexpressing (**Methods**) and then performed differential gene expression and gene set enrichment analysis (GSEA). In accordance with the role of *ADSL* in *de novo* DNA synthesis, we found an upregulation of genes involved in the purine nucleoside biosynthetic process pathways as well as DNA replication and repair in the *ADSL*-overexpressing samples (p adj = 0.025; **Figure 3A** and**Figure S4A-B**). We found that expression of the 'replicative' DNA polymerase subunits *POLA2, POLD2,* and* POLE2* genes, as well as the proliferative marker *PCNA* correlated with *ADSL* expression (**Figure S4B**). Genes involved in the firing of DNA replication (*CDC45, MCM2, CDT1*) also showed significant positive correlation with *ADSL* in CRCs (**Figure S4B**). Of note, we found enrichment of genes involved in DNA repair, and specifically DNA repair-associated DNA synthesis (**Figure 3A** and**Figure S4C**). In particular, S-phase checkpoint genes involved in the downstream protection of stalled DNA replication forks (e.g. *RAD51*) as well as S-phase signaling mediators (e.g. *CHEK2*), previously reported to be upregulated in CRCs, showed significant positive correlation with *ADSL* expression (**Figure S4C**) 40. We therefore tested whether forced expression of ADSL might drive DNA replication stress in CRC by immunostaining H2AX phosphorylation at serine 139 (γH2AX), a sensitive indicator of both DNA damage and DNA replication stress 41. Indeed, *ADSL*-silenced DLD-1 cells showed weaker staining for γH2AX compared to control cells, while forced expression of *ADSL* in Caco-2 strongly upregulated γH2AX protein staining (**Figure 3B**). *ADSL*-overexpressing CAM tumors also showed higher expression of the DNA damage marker γH2AX (**Figure 3C** and**Figure S5A**), further suggesting a role of ADSL in DNA replication stress.

Pathways related to cell cycle, specifically G1 to S phase transition, were also significantly upregulated in *ADSL*-overexpressing versus non-*ADSL*-overexpressing CRCs (p adj = 0.025; **Figure 3A** and**Figure S4D**)**.** We found that *ADSL* expression correlated with many cyclins and cell-cycle checkpoints related genes. We also observed a correlation between the expression levels of *ADSL* and the *MYC* oncogene, whose expression has been shown to be indirectly regulated by ADSL in triple-negative breast cancer 6 and whose upregulation of target genes is a signature of the CMS2 subtype 37. In accordance with the enrichment in the CMS2 molecular subtype, *ADSL*-overexpressing tumors also showed significant enrichment of the canonical WNT signaling pathway (p = 0.037; **Figure 3A**).

Given the role of *ADSL* in *de novo* DNA synthesis and that the cell cycle pathway, specifically G1 to S phase transition, was also significantly upregulated in *ADSL*-overexpressing CRCs, we hypothesized that modulation of *ADSL* expression might also affect cell cycle in CRC cells. We therefore assessed the distribution of cells in the different cell-cycle phases upon modulation of* ADSL* expression by flow cytometry (FACS) (**Figure S5B**). Under normal growth conditions (complete cell culture medium), transient *ADSL* knock-down showed a tendency to increase the percentage of DLD-1 and SW480 cells in the S-phase, while *ADSL* overexpression significantly increased the fraction of Caco-2 and HT-29 cells in the G1 phase (**Figure 3D** and**Figure S5C**). To determine if the accumulation of *ADSL*-overexpressing cells in the G1 phase was a result of a block in G1 phase or an indicator of faster cell cycle and increased proliferation rate, we synchronized cells at the G1-S transition checkpoint by glucose deprivation 42 and again analyzed cell cycle upon modulation of *ADSL* expression. Given the key role of *ADSL* in *de novo* purine synthesis and glucose being the primary substrate of this metabolic pathway, we expected to have a stronger phenotype upon glucose restriction. Indeed, under glucose-depleted growth conditions, *ADSL*-silenced DLD-1 and SW480 cells could not proceed from S to G2/M phase, which resulted in significant accumulation of cells in S phase compared to control cells, while no significant difference in the proportion of cells in the S phase was observed between *ADSL*-silenced and control cells in full medium (**Figure 3E** and**Figure S5D**). By contrast, *ADSL* overexpression facilitated cell transition from S-phase to G2/M phase in both Caco-2 and HT-29 CRC cells (**Figure 3E** and**Figure S5D**).

Together with the exploratory analysis performed on the TCGA cohort, our data suggest the involvement of ADSL in the deregulation of cell cycle and DNA repair/replication mechanisms during the process of colorectal oncogenesis. In particular, our results show that ADSL overexpression accelerates progression through the cell cycle (**Figure 3F**).

### ADSL expression levels predict response to 6-mercaptopurine in *in vitro*, *in vivo* and in patient-derived organoids

**Figure 4:**
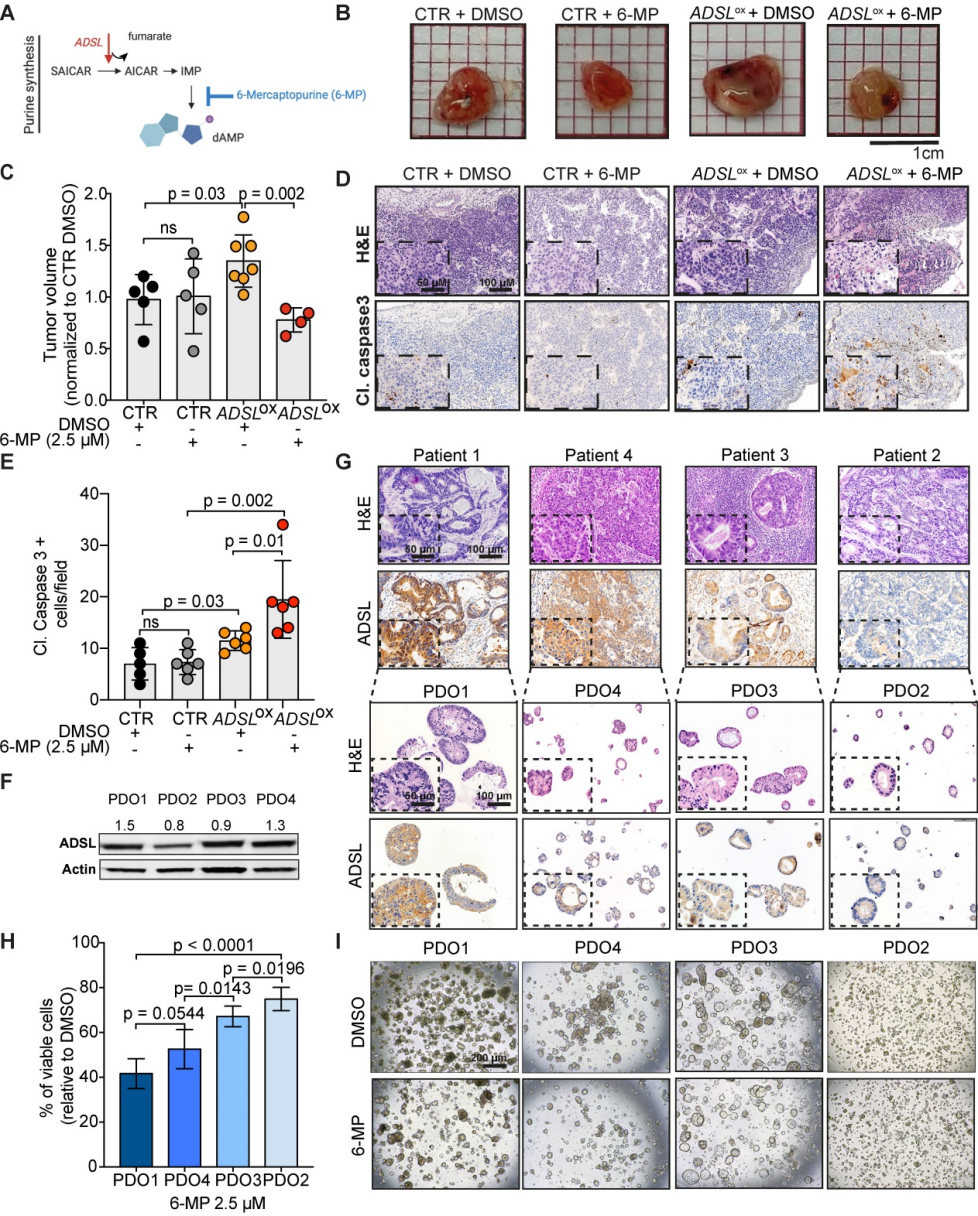
**ADSL expression levels predict response to 6-mercaptopurine in *in vitro*, *in vivo* and PDOs.** (**A**) Schematic diagram of the role of *ADSL* as well as 6-MP inhibitory effect in the *de novo* purine biosynthetic process. (**B**) From the left: pictures of Caco-2 control cells pre-treated with DMSO, Caco-2 control cells pre-treated with 6-MP, *ADSL-*overexpressed Caco-2 cells pre-treated with DMSO and *ADSL-*overexpressed Caco-2 cells pre-treated with 6-MP implanted in CAMs and grown for 4 days post-implantation. (**C**) Quantification of tumor growth derived from the CAM experiment (n = at least 4 tumors from 2 independent experiments); mean ± SD. Values are normalized to the mean of CTR DMSO. (**D**) Representative micrographs of Caco-2 tumors extracted 4 days post-implantation. Tissue sections were stained with Hematoxylin-eosin (H&E) and immunostained with the apoptotic marker cleaved Caspase 3 in the different treatment conditions. (**E**) Quantification of cleaved caspase 3 positive cells in the tumor from (**C**). (**F**) Immunoblot showing ADSL protein expression in the different CRC-PDOs. Quantification is relative to the loading control (Actin). (**G**) Representative pictures of matched tissue-organoids pairs. Both tissues and organoids sections were stained with Hematoxylin-eosin (H&E) (upper panel) and ADSL antibody (lower panel). (**H**) Percentage of viable cells relative to DMSO in CRC-PDOs treated with 2.5 µM of 6-MP. (**I**) Representative pictures of the organoid growth after 5 days treatment with 2.5 µM of 6-MP. Data are mean ± SD (**B**,** E**,** H**) n ≥ 3 replicates. For all experiments, statistical significance was assessed by unpaired t-test. Scale bars are 50 and 100 µm for (**D**, **G**) and 200 µm for (**I**).

The antimetabolite 5-FU is the most commonly used chemotherapeutic agent for CRC treatment. To evaluate whether ADSL expression could modulate response to 5-FU, we compared the viability of Caco-2 *ADSL*-overexpressing and control cells upon treatment with 5-FU. *ADSL* overexpression did not significantly affect response to 5-FU *in vitro* (**Figure S6A-B**). 5-FU is, however, a pyrimidine analogue, while ADSL is involved in the conversion of the intermediate molecule SAICAR into AICAR and fumarate in the purine nucleotide cycle (**Figure 4A**). We therefore asked whether *ADSL* expression levels may instead better predict response to a purine analogue antimetabolite, such as 6-mercaptopurine (6-MP). 6-MP competes with inosine monophosphate (IMP) thus inhibiting the same biosynthetic pathway in which ADSL plays a role (**Figure 4A**). As hypothesized, *ADSL* overexpression significantly sensitized Caco-2 cells to 6-MP (p < 0.001; **Figure S6C-D**). Caco-2 cells overexpressing *ADSL* treated with 6-MP also showed positivity for the apoptotic marker cleaved caspase 3 (**Figure S6E**)**.** To determine whether modulating *ADSL* expression levels would also affect response to 6-MP *in vivo,* we pre-treated *ADSL*-overexpressing Caco-2 cells with 6-MP for 24 h before implantation in the CAM and then screened for tumor formation. In accordance with our results obtained *in vitro*, treatment with 6-MP significantly reduced tumor volume only upon *ADSL* overexpression (**Figure 4B**). Indeed, no significant difference could be appreciated when comparing the tumors derived from control cells treated with DMSO or 6-MP, while the volume of tumors derived from *ADSL*-overexpressing cells pre-treated with 6-MP was significantly smaller compared to *ADSL*-overexpressing cells pre-treated with DMSO (**Figure 4C**), suggesting that *ADSL* expression also modulates response to 6-MP *in vivo*. *ADSL-*overexpressing tumors pre-treated with 6-MP also showed a significantly stronger positive signal for cleaved caspase 3, as well as morphological features of apoptosis in H&E staining (e.g. hypereosinophilic cytoplasm, nuclear fragmentation, “apoptotic bodies”, **Figure 4D-E** and**Figure S7**), demonstrating that 6-MP induces apoptosis more efficiently in *ADSL*-overexpressing CRCs and supporting the importance of an efficient purine nucleotide cycle for the oncogenic activity mediated by ADSL.

Accumulating evidence indicates that CRC-patient derived organoids (PDO) retain molecular features of the original tumor, and they are amenable to drug screening and response prediction in a preclinical setting 43-45. We therefore decided to explore whether differences in ADSL level may predict response to 5-FU or 6-MP in four PDOs generated from liver metastasis of CRC patients (PDO1 to PDO4; **Table S3**). The PDOs showed different levels of ADSL protein expression by immunoblot (**Figure 4F**). Immunohistochemical analysis of ADSL showed cytoplasmic immunoreactivity in all original tissues and their derived organoids, with ADSL expression levels that were consistent with the immunoblot results (**Figure 4G**). All the PDOs retained the histopathological features of the original tumor tissue from which they were derived (**Figure 4G** and**Figure S8**). Indeed, hematoxylin and eosin (H&E) staining confirmed that tumor-derived organoids resembled the patient-specific morphology heterogeneity, ranging from cystic to solid/compact phenotype (**Figure 4G**), as well as a similar staining pattern for colorectal-specific markers (**Figure S8**).

Using these CRC-PDOs, we explored whether differences in ADSL level may affect response to 5-FU or 6-MP in a pre-clinical setting. Contrary to the results obtained* in vitro*, 5-FU treatment had a moderate but significant effect in reducing cell viability in ADSL-high PDOs (PDO1 and PDO4) compared to ADSL-low PDOs (PDO2 and PDO3; **Figure S9A**), suggesting that ADSL may also be partially involved in the cell response to 5-FU. On the other hand, consistent with our *in vitro* results, 6-MP treatment on PDOs demonstrated a strong ADSL-level-dependent response. In fact, PDOs with high ADSL expression (PDO1 and PDO4) showed higher sensitivity to 6-MP compared to PDOs with lower ADSL expression (PDO2 and PDO3) in terms of cell viability (**Figure 4H**) and cell proliferation (**Figure 4I**). To test whether 6-MP and 5-FU may act synergistically in CRC, we treated PDO1 and PDO3, respectively with high and low ADSL expression, with different dosages of 5-FU alone or in combination with a fixed dose of 6-MP. While the addition of 6-MP to 5-FU significantly reduced the percentage of cell viability in PDO1, the difference between treatment with 6-MP alone or in combination with 5-FU was minor (approximately 60% viable cells with 6-MP alone versus 50% in combination with all three concentrations of 5-FU; **Figure S9B**, left). On the contrary, PDO3 was resistant to both treatments alone or in combination (**Figure S9B**, right). Overall our data do not support the synergistic effect of 6-MP and 5-FU and suggest that ADSL overexpression may specifically sensitize CRCs to treatment with 6-MP.

### ADSL overexpression causes mitochondrial dysfunction and oxidative stress

**Figure 5:**
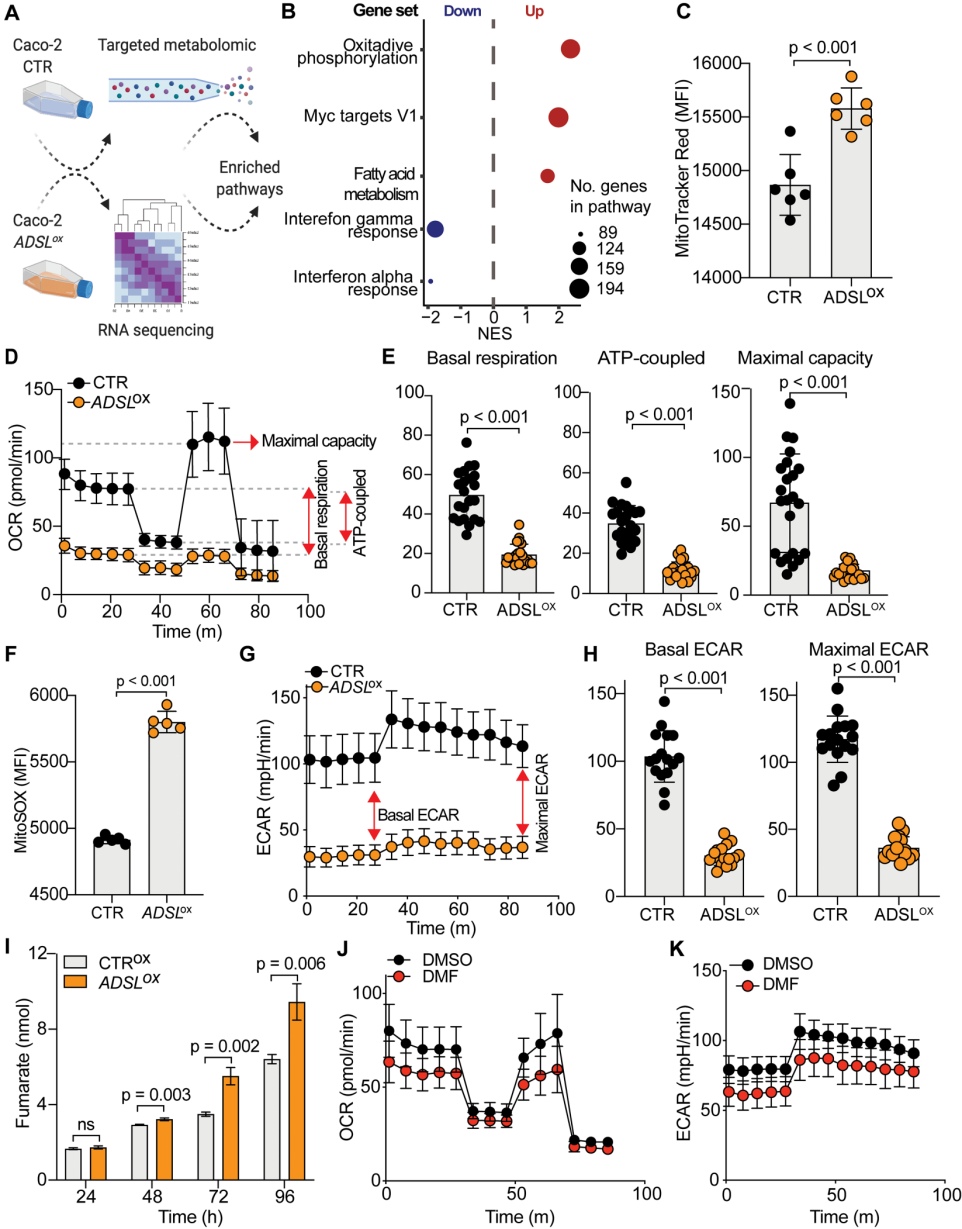
**ADSL overexpression causes mitochondrial dysfunction and oxidative stress.** (**A**) Schematic representation of the multi-omics approaches performed on Caco-2 cells control and overexpressing ADSL. (**B**) Normalized enrichment scores of significantly up- and down-regulated pathways identified by gene set enrichment analysis in control and *ADSL*-overexpressing Caco-2 cells. (**C**) Mitochondrial membrane potential measured by flow cytometry in control and ADSL overexpressing Caco-2 cells stained with MitoTracker Red. Y-axis represents mean fluorescence intensity (MFI). (**D**) Oxygen-consumption rate (OCR) of control and *ADSL*-overexpressing Caco-2 cells in real time under basal conditions and after drug-induced mitochondrial stress 73 with oligomycin, FCCP and rotenone plus antimycin A. (**E**) Mean basal, ATP-coupled and maximal respiratory capacity of the cells in (**D**). (**F**) Mitochondrial reactive oxygen species measured by flow cytometry in control and *ADSL*-overexpressing Caco-2 cells stained with MitoSOX. Y-axis represents mean fluorescence intensity (MFI). (**G**) ECAR of control and *ADSL* overexpressing Caco-2 cells in real time after treatment as in **D**. (**H**) Mean basal and maximal ECAR of the cells in **D**. (**I**) Extracellular fumarate level (nmol) in control and *ADSL*-overexpressing Caco-2 cells 24, 48, 72 and 96 h post transfection. (**J**,** K**) OCR (**J**) and ECAR of parental Caco-2 cells treated with DMSO or fumarate (50 µM) in real time after treatment as in **D**.

To understand the molecular basis of oncogenic properties of ADSL we performed targeted metabolomic and global transcriptomic analysis on *ADSL*-overexpressing and control Caco-2 cells (**Figure 5A**). In line with the well-defined role of ADSL, *ADSL-*overexpressing cells showed a significant fold increase in metabolites involved in purine as well as pyrimidine biosynthetic processes (**Figure S10A**). Likewise, metabolites from mitochondrial tricarboxylic acid (TCA) cycle and beta-oxidation were also significantly enriched (**Figure S10A**). Mirroring the metabolomic data, gene set enrichment analysis performed on the transcriptomic data showed that oxidative phosphorylation and lipid metabolism were indeed dysregulated in *ADSL*-overexpressing cells, together with the up-regulation of Myc targets (**Figure 5B**). Given that mitochondrial processes such as oxidative phosphorylation and TCA cycle were significantly dysregulated in both omics' analyses, we hypothesized a role for ADSL in regulating mitochondrial function in CRC. Indeed, *ADSL*-overexpressing CRCs from the TCGA cohort showed an enrichment in genes involved in oxidative phosphorylation and mitochondrial respiratory chain (**Figure 3A**). To further explore this hypothesis, we measured the mitochondrial membrane potential of *ADSL*-overexpressing and control cells using MitoTracker Red. In line with the up-regulation of oxidative phosphorylation seen at the gene expression level, *ADSL-*overexpressing cells showed significantly higher membrane potential (**Figure 5C**). However, this was not reflected in a higher mitochondrial respiration rate (**Figure 5D**). By contrast, *ADSL-*overexpressing cells showed significantly reduced oxygen consumption rate (OCR) compared to control cells (**Figure 5D**). Specifically, *ADSL* overexpression significantly impaired both basal and ATP-coupled respiration as well as maximal respiratory capacity in Caco-2 cells (**Figure 5E**). Leak respiration and non-mitochondrial respiration were also significantly reduced (**Figure S10B**).

Previous studies have demonstrated that generation of reactive oxygen species (O2(*-), ROS) is partially regulated by the membrane potential (Δψm), such that an increase in mitochondrial membrane potential due to dysfunctional electron transport favors ROS production 46. Indeed, *ADSL* overexpression significantly increased mitochondrial ROS production in Caco-2 cells, as measured using MitoSOX staining (**Figure 5F**).

Reduced aerobic respiration is usually accompanied by a higher glycolytic rate in cancer cells 47. However, *ADSL-*overexpressing cells additionally showed a significantly lower basal and maximal glycolytic capacity (**Figure 5G-H**). Accordingly, we also demonstrated that the impact of *ADSL* overexpression on cell cycle progression was significantly greater upon glucose depletion (**Figure 3D**).

ADSL catalyzes the conversion of SAICAR to AICAR as well as S-AMP to AMP, and in both reactions fumarate is generated. Fumarate is a TCA cycle intermediate and a well-known oncometabolite which has been connected to ADSL oncogenicity in endometrial cancer 7. Additionally, fumarate accumulation has been shown to cause respiratory chain defects 48 as well as to impair glycolysis 49. Accordingly, we found that *ADSL* overexpression led to a significant fumarate accumulation in Caco-2 cells in a dose-dependent manner (**Figure 5I** and**Figure S10C**). Treating parental cells with dimethyl fumarate (DMF), a cell-permeable form of fumarate, significantly impared cellular respiration, albeit to a lesser extent compared to *ADSL* overexpression (**Figure 5J**). Specifically, DMF significantly impacted basal and ATP-coupled respiration of Caco-2 cells, whilst there was no observable effect on maximal respiratory capacity and leak respiration (**Figure S10D**). Similarly, DMF significantly impaired the glycolytic activity of parental Caco-2 cells (**Figure 5K**), both at their basal and maximal capacity (**Figure S10E**).

### The KEAP1-NRF2 and the mTOR-cMYC axis are independently activated upon* ADSL* overexpression in CRC

**Figure 6:**
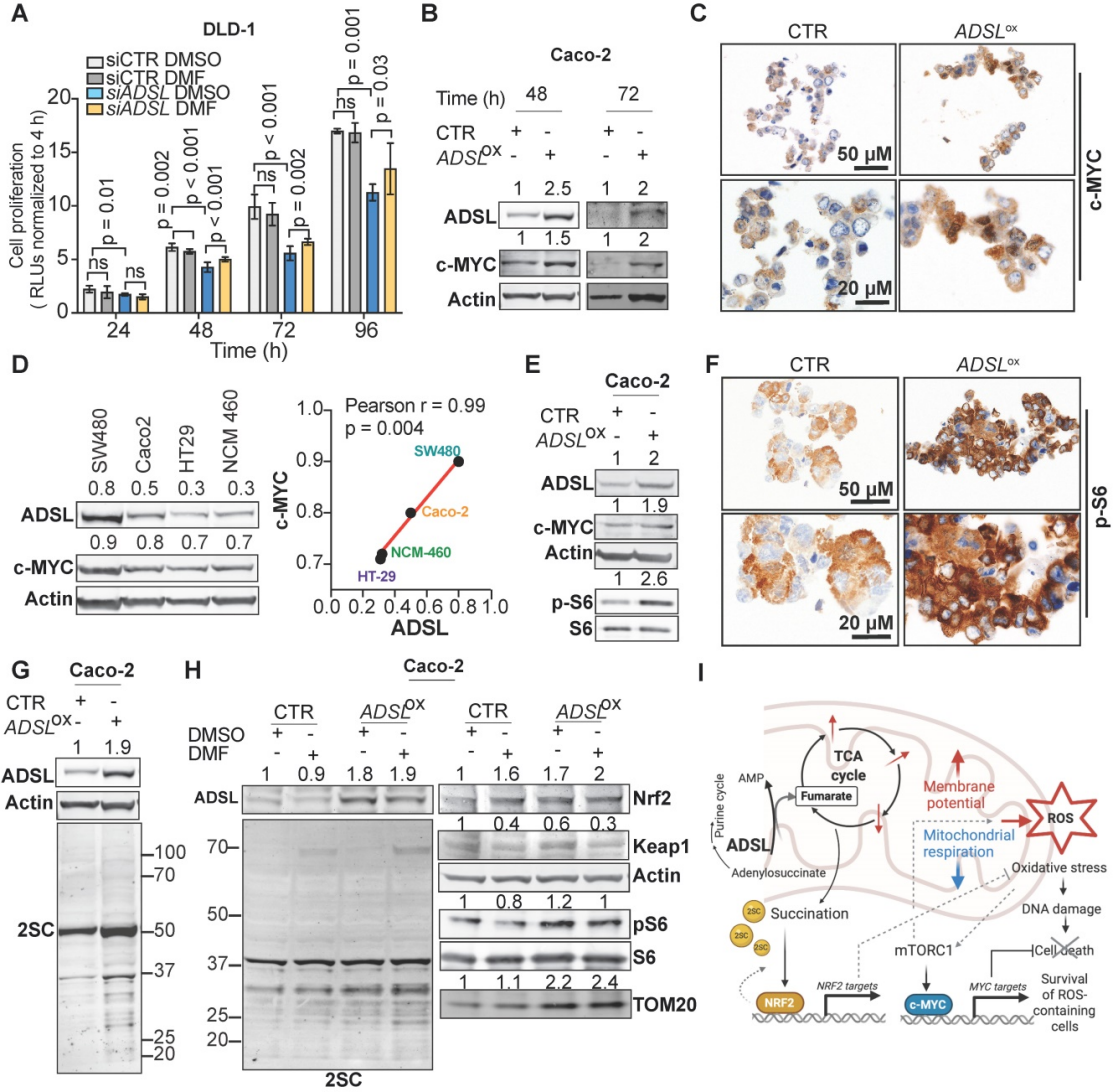
**ADSL overexpression induces succination and mTOR activation and increases c-MYC protein expression.** (**A**) Proliferation capacity of DLD-1 control and *ADSL*-silenced cells treated with DMSO or fumarate (50 µM). (**B**) Immunoblot showing ADSL and c-MYC expression in the Caco-2 cells at 48 and 72 h post transfection. (**C**) Representative pictures of control and *ADSL*-overexpressing Caco-2 cells immunostained with c-MYC. Scale bars 20-50 µm. (**D**) Immunoblot showing ADSL and c-MYC expression in CRC cell lines (left). Correlation (linear regression) between c-MYC (Y-axis) and ADSL (X-axis) levels of expression (relative to actin). (**E**) Immunoblot showing ADSL, c-MYC and phospho and total S6 expression in Caco-2 cells 48 h post-transfection. (**F**) Representative pictures of control and *ADSL*-overexpressed Caco-2 cells immunostained with phospho-S6 (p-S6). Scale bars 20-50 µm. (**G**) Immunoblot showing ADSL and succination (2-SC) levels in Caco-2 cells 48 h post-transfection. (**H**) Immunoblot showing ADSL and succination (2-SC) levels (left) and KEAP1, NRF2, TOM20, total and phospho-S6 in control and *ADSL*-overexpressing Caco-2 cells treated with DMSO or fumarate (50 µM). (**I**) Schematic representation of ADSL-driven pro-oncogenic effects in CRC cells.

To test the causality between fumarate increase and the phenotype driven by ADSL dysregulation, we performed a rescue experiment by adding DMF to DLD-1 cells after transfection with siRNAs against *ADSL* or control siRNAs. The addition of DMF significantly increased the proliferation rate of *ADSL*-silenced cells (**Figure 6A**). However, fumarate only partially rescued the phenotype induced by *ADSL* gene knock-down. We therefore asked which other molecular mechanism could account for the pro-proliferative role of *ADSL* in CRC. Given that *ADSL*-overexpressing Caco-2 cells displayed a significant enrichment for *MYC*-targets (**Figure 5B** and **Figure S11A**) and that *ADSL* was previously shown to activate the c-MYC pathway in triple negative breast cancer 6, we therefore asked whether a similar mechanism occurs in CRC.

While *MYC* mRNA levels only mildly increased upon *ADSL* overexpression (**Figure S11B**), we detected a 1.5- and 2-fold increase in the c-MYC protein levels in *ADSL*-overexpressing Caco-2 cells at 48 and 72 h post-transfection, respectively (**Figure 6B**). The increase in c-MYC protein was further detected by immunohistochemistry (**Figure 6C**) and similar results were also obtained in the HT-29 cell line (**Figure S11C-D**). Accordingly, c-MYC and ADSL protein levels significantly and positively correlated in CRC cell lines (Pearson r = 0.99, p = 0.004; **Figure 6D**). Supporting our *in vitro* data, *ADSL*-high tumors are significantly enriched in the CRC molecular subtype CMS2 (**Figure S2A**), characterized by a strong upregulation of *MYC* targets.

Next, we asked through which mechanism *ADSL* overexpression was able to induce c-MYC. It was previously shown that ADSL levels modulate Akt phosphorylation in endometrial cancer and that TCA cycle defects, specifically the accumulation of α-ketoglutarate (αKG), as well as ROS increase can activate the mTOR signaling pathway 7,50-53. mTORC1, a major regulator of cellular metabolism and energetic state, has been shown to regulate *MYC* mRNA translation 54,55. Here we observed that S6 phosphorylation, a marker of mTORC1 activation, increased upon *ADSL* overexpression in both Caco-2 and HT-29 cells (**Figure 6E-F** and **Figure S11E**). Similarly, *ADSL* knock-down reduced phospho-S6 and c-MYC levels in DLD-1 cells (**Figure S11F**).

Fumarate has been shown to modify thiol groups in several proteins by forming *S*-(2-succinyl) Cys (2SC) adducts, a process termed as succination 56. Succination of KEAP1, a repressor of the transcription factor nuclear factor, erythroid 2 like 2 (NFE2L2/NRF2), is known to promote NRF2 stabilization and nuclear translocation 57, with consequent induction of stress response genes 57-59. Using an anti-2SC antibody we tested whether *ADSL* overexpression, via increasing Krebs cycle intermediates such as fumarate, would increase succination in CRC cells. Indeed, we detected higher 2SC levels in *ADSL-*overexpressing Caco-2 cells compared to control (**Figure 6G**), while silencing *ADSL* reduced succination to the endogenous level (**Figure S11G**). Modulation of *ADSL* had similar effects on 2SC levels in DLD-1 cells (**Figure S11H**). The addition of fumarate also increased 2SC levels in Caco-2, however the succination pattern slightly differed from that induced by *ADSL* overexpression (**Figure 6H,** left panel). As expected, 2SC levels correlated with reduced KEAP1 levels and NRF2 upregulation upon both the addition of fumarate and *ADSL* overexpression (**Figure 6H,** right panel). However, fumarate alone did not induce S6 phosphorylation nor c-MYC up-regulation, indicating that ADSL overexpression activates the mTOR-MYC axis through mechanisms other than fumarate accumulation (**Figure 6H** and**Figure S11I**). Additionally, only *ADSL* overexpression and not fumarate increased the expression of the mitochondrial outer membrane protein TOM20, a common marker of mitochondrial mass and/or biogenesis (**Figure 6H**), which is also a typical downstream effect mediated by c-MYC activation 60.

Taken together our data indicate that ADSL overexpression independently activates the NRF2 stress response pathway and the mTORC1-cMYC axis. Activation of both NRF2 and mTORC1-cMYC may help protect CRC cells from oxidative stress while allowing cell survival and proliferation of DNA damage-prone tumor clones (**Figure 6I**).

## Discussion

In the present study, we identified ADSL as a novel putative oncogene in CRC. ADSL is a key enzyme of *de novo* purine biosynthesis 4,5. Increased levels of ADSL have been observed in several cancer types 6-8 and have been shown to increase proliferation, migration, and invasive capability of endometrial and triple-negative breast cancer cell lines 6,7. Here, we have demonstrated that ADSL is upregulated in CRC at both the mRNA and protein levels. However, while our TMA cohort of CRC primary tumors suggested that ADSL expression was reduced in advanced-stage tumors, we did not observe any difference in ADSL expression in our second TMA cohort consisting of primary CRC tumors and CRC metastases. On the mRNA level, *ADSL* expression levels also showed a downward trend with increasing stages, albeit with very small differences in expression levels between tumors of different stages. Although it is unclear whether ADSL levels are genuinely reduced in advanced-stage CRCs, our results suggest that ADSL plays an oncogenic role in CRC initiation rather than tumor progression, which would be consistent with the upregulated *ADSL* activity in colon pre-neoplastic lesions 15.

By modulating the expression levels of *ADSL in vitro,* we showed that *ADSL* promotes proliferation and migration in CRC cells, as well as tumor growth *in vivo* in the CAM model. Additionally, we showed that ADSL regulates cell cycle progression. Indeed, silencing of *ADSL* blocked cells in the S-phase while upon forced expression of *ADSL*, cells progressed faster through the cell cycle. Accordingly, *ADSL*-overexpressing CRCs showed overexpression of DNA synthesis and cell-cycle related pathways. In particular, we found that *ADSL* overexpression correlates with the expression of many genes coding for DNA polymerases and S-phase signaling checkpoints, many of which are part of a “DNA replication signature” found dysregulated in colorectal cancer 40. These findings demonstrate the oncogenic effect of *ADSL* and emphasize that* ADSL* may be a potential therapeutic target in CRC.

The pyrimidine analogue 5-FU is one of the most commonly used chemotherapeutic agents for CRC 9,13,61. Given the essential role of *ADSL* in DNA synthesis and cell cycle, we investigated its potential as a predictive biomarker of response to 5-FU *in vitro* and *ex vivo*. *ADSL* overexpression did not significantly affect response to 5-FU in CRC cells, but it partially affected response in CRC-PDOs. The discordant results may reflect the differential cellular response between traditional two-dimensional cell culture models derived from single clones compared to PDOs that better represent tumor heterogeneity 62, underlining the importance of using multiple preclinical models for accurate drug-response prediction. The pivotal role played by *ADSL* in *de novo* purine biosynthesis led us to hypothesize that a purine analogue rather than a pyrimidine analogue may be more effective on *ADSL*-overexpressing CRCs. Indeed, our data strongly indicate that *ADSL* overexpression significantly sensitizes CRCs to 6-MP both *in vitro* and *in vivo*. Additionally, we showed that CRC-PDOs with high ADSL expression respond better to 6-MP compared to ADSL-low PDOs. As a chemotherapeutic agent, 6-MP is used in the treatment of acute lymphoblastic leukemia 63. In the context of gastrointestinal diseases, 6-MP as an immunosuppressant is one of the cornerstones of treatment in inflammatory bowel diseases 64,65, but there is a substantial lack of studies on the use of 6-MP in chemotherapy regimens in CRC. To our knowledge, only one study has previously investigated the potential use of 6-MP as a chemotherapeutic agent in CRC 66, with no detection of any substantial clinical benefit. We speculate that patient stratification based on *ADSL* expression may help better dissect the potential benefit of 6-MP in the treatment of CRC. Unfortunately, due to the lack of clinical studies evaluating the effect of 6-MP in CRC patients, we cannot draw any conclusion regarding the predictive value of ADSL expression in response to 6-MP treatment in the overall survival or disease-free survival in patients.

Using targeted metabolomics and transcriptomics analysis we showed that ADSL overexpression mainly affects mitochondrial function, leading to the accumulation of Krebs cycle intermediates and altered oxidative phosphorylation. In particular, we demonstrated that ADSL overexpression lowered the oxygen consumption rate (OCR) in CRC cells. Reduced cellular respiration is a common strategy used by cancer cells to survive in hypoxic conditions 67. Although impairment in oxidative metabolism is usually associated with increased glycolysis in cancer 67, we found that ADSL overexpression impairs glycolysis as well, thus suggesting that other metabolic pathways might fuel the energetic and anabolic demands of *ADSL*-overexpressing CRC cells, such as fatty acid metabolism that was also found significantly up-regulated upon *ADSL* overexpression.

Fumarate is a TCA cycle intermediate and a product of ADSL catalyzed reactions. Fumarate has been shown to inhibit both mitochondrial respiration 48 and glycolysis 49, as well as to mediate ADSL oncogenic properties in other cancer types 7. Although the forced expression of *ADSL* increased fumarate abundance which we postulated contributed to the significant impairment of mitochondrial respiration and glycolysis in the parental CRC cells, treatment with exogenous fumarate only partially mimicked the metabolic phenotype induced by *ADSL* overexpression. Our data indicate that dysregulation of additional metabolites or target genes is required for the full oncogenic potential of *ADSL* in CRC. Indeed, we also found that *ADSL* overexpression, but not fumarate, induces mTORC1 activation and c-MYC protein increase in CRC cell lines. Our results are in line with the current literature reporting that ADSL indirectly modulates Akt phosphorylation and c-MYC activation in other cancer types 6,7. Both mTOR and c-MYC are well-known master regulators of cellular metabolism and have been shown to regulate the TCA cycle and mitochondrial function, thus driving metabolic rewiring in cancer 68-70. In particular, both mTOR and c-MYC can stimulate the synthesis of mitochondria-targeted proteins and promote mitochondrial biogenesis 60,70, which could partially explain the upregulation of nuclear-encoded mitochondrial genes. Decreased mitochondrial respiration coupled with increased membrane potential can result in ROS accumulation. Indeed, *ADSL* overexpression increased mitochondrial ROS levels in Caco-2 cells and triggered the accumulation of DNA-damage, as proved by the overexpression of γH2AX. Given that defects in both the TCA cycle and ROS accumulation can activate the mTOR signaling pathway 7,50-53, we speculate that, upon *ADSL* overexpression, the accumulation of ROS leads to the activation of the mTORC1-cMYC axis. mTOR and c-MYC in turn induce mitochondrial biogenesis through a positive feedback loop which exacerbates the phenotype. Our current data cannot discriminate whether the c-MYC increase is induced by mTORC1 activation or vice versa, as well as to what extent mitochondrial dysfunction with subsequent ROS generation causes, or is caused by, the activation of the mTORC1-cMYC pathway. Additional experiments are required to elucidate in more detail how *ADSL* overexpression drives these mechanisms.

Fumarate accumulation is also known to cause protein succination. Succination of regulatory proteins such as KEAP1 and p62, causes the activation of the NRF2-mediated response, which promotes tumorigenesis by enhancing ROS detoxification. Indeed, both fumarate and *ADSL* overexpression induced higher levels of protein succination as well as the activation of the KEAP1-NRF2 pathway. While the KEAP1-Nrf2 pathway might appear as an independent pathway, the mTORC1-cMYC and the KEAP1-Nrf2 pathway are tightly interconnected and act synergistically in cancer initiation driven by ROS accumulation. Indeed, it has been shown that activation of both NRF2 stress-response and mTORC1-cMYC axis are required for the survival and expansion of ROS-abundant cells in the early stages of hepatocarcinogenesis 71. Additionally, while NRF2 is a regulator of redox homeostasis in quiescent cells, in the presence of an active PI3K-Akt pathway, it can also drive the expression of genes involved in glutamine metabolism and the pentose phosphate pathway 72. We therefore hypothesized that by inducing mitochondrial dysfunction and ROS generation, ADSL acts as an oncogene in CRC by up-regulating both NRF2 and mTORC1-cMYC axis.

In conclusion, our investigation highlights the multifaceted role of *ADSL* as a new oncogene in colorectal cancers and strongly supports a role for *ADSL* overexpression in sensitizing tumor cells to 6-MP. Specifically, we demonstrated that *ADSL* overexpression in CRC: 1) induces dysregulation of the Krebs cycle and mitochondrial dysfunction, with consequent 2) activation of the mTOR-cMYC pathway, and 3) NRF2 stress response. Our results show that ADSL overexpression is pleiotropic in CRC, in the sense that it induces metabolic and mitochondrial dysfunction with consequent oxidative stress and ROS accumulation via a series of interconnected pathways, which favors the survival of stressed CRC cells and enable the accumulation of DNA damage.

## Supplementary Material

Supplementary figures and tables.

## Abbreviations

2SC: *S*-(2-succinyl) Cys
5-FU: 5-fluorouracil
6-MP: 6-mercaptopurine
ADSL: adenylosuccinate lyase
AICAR: 5-Aminoimidazole-4-carboxamide-1-β-d-ribofuranoside
AJCC: American Joint Committee on Cancer
CAM: chicken chorioallantoic membrane
CIN: chromosomal instability
Cl. Caspase: cleaved caspase
CMS: consensus molecular subtype
CRC: colorectal carcinoma
DAB: 3,3′-Diaminobenzidine
DAPI: 4′,6-diamidino-2-phenylindole
DAPI-A: 4′,6-diamidino-2-phenylindole-area
dAMP: deoxyadenosine monophosphate
DMEM: Dulbecco's Modified FEagle Medium
DMSO: dimethyl sulfoxide
DRIVE: deep RNAi interrogation of viability effects in cancer
FACS: fluorescence-activated cell sorting
FBS: fetal bovine serum
FDR: false discovery rate
FSC-A: forward scatter area
FSC-W: forward scatter width
GAPDH: glyceraldehyde 3-phosphate dehydrogenase
GSEA: gene set enrichment analysis
H&E: hematoxylin and eosin
IHC: immunohistochemistry
IMP: inosine monophosphate
KRT20: keratin 20
KLRC3: natural killer cells lectin-like receptor C3
NES: normalized enrichment score
PAS: periodic acid-Schiff
PCR: polymerase chain reaction
PBS: phosphate-buffered saline
PDO: patient-derived organoids
PFA: Paraformaldehyde
PI: propidium iodide
RTCA: Real-Time Cell Analyzer
RT-PCR: reverse transcription polymerase chain reaction
SAICAR: phosphoribosylaminoimidazolesuccinocarboxamide
SD: standard deviation
SSC-A: side scatter-area
TCGA: The Cancer Genome Atlas
TMA: tissue microarray
TRG: tumor regression grade
