# Supplementary material for: Adenylosuccinate lyase is oncogenic in colorectal cancer by causing mitochondrial dysfunction and independent activation of NRF2 and mTOR-MYC-axis
Source: Theranostics. 2021 Feb 15;11(9):4011–29. doi: 10.7150/thno.50051 (PMC7977451; doi:10.7150/thno.50051)
Supplement: Supplementary file 1 — Supplementary figures and tables. [file thnov11p4011s1.pdf]

Supplementary Methods

Tissue Microarray (TMA)

Tissue cylinders with a diameter of 1 mm were punched from morphologically representative areas of each donor block and brought into one recipient paraffin block (30x25 mm) using the TMA GrandMaster® (TMA-GM; 3D-Histech Ltd, Sysmex AG, Switzerland) technology. Each punch was derived from the center of the tumor in an area with no necrosis so that each TMA spot consisted of more than 50% tumor cells.

Tumor-stromal ratio

For all tumors, the tumor-stromal ratio on 4 µm H&E stained tissue sections was calculated as described previously [1] and the stromal percentage was estimated per 10% interval. Tumors were divided into stroma-high (>50%) and stroma-low (≤50%) groups according to their highest score.

Microsatellite Instability (MSI)

Immunohistochemical analyses of mismatch repair proteins were performed for expression of the four mismatch repair proteins MLH1, MSH2, MSH6, and PMS2 as previously described [2]. Tissue samples with tumor cells lacking nuclear staining for at least one of these proteins were considered to have a positive MSI screening status, hereafter referred to as MSI. Negative MSI screening status based on immunohistochemical staining is hereafter referred to as microsatellite-stable (MSS).

Analysis of The Cancer Genome Atlas (TCGA) dataset

FPKM gene-level expression and raw read counts data for TCGA CRC cohort [3] with 622 tumors and 51 non-tumoral tissues, defined as “Solid Tissue Normal”, were obtained from TCGA Genomics Data Commons harmonized data portal using TCGAbiolinks R package [4].

The expression of ADSL was compared between tumors and normal tissues using the student's t-test. Tumor samples were classified into ADSL-overexpressing (n=218) and non-ADSL-overexpressing (n=404) groups based on the threshold of mean + 2 standard deviations of normal tissues. Clinical information was obtained from the Human Protein Atlas (Pathology Atlas) [5] CRC project for 596 TCGA CRCs. Differential expression analysis was performed between ADSL-overexpressing and non-ADSL-overexpressing groups using the edgeR package [6]. Genes with low expression (<1 log-counts per million in ≥ 50 samples) were filtered out. Normalization was performed using the "TMM" (weighted trimmed mean) method [7] and differential expression was assessed using the quasi-likelihood F-test. Gene set enrichment analysis was performed using the fgsea [8] package with genes ranked based on signed p-value according to the direction of the log-fold change. Gene Ontology (GO) gene sets from MSigDB [9] were used to identify significantly upregulated/downregulated pathways. Molecular subtyping was performed using the CMScaller package [10].

Cell culture

Mammalian cells were maintained in a 5% CO_2-humidified atmosphere at 37°C. Colon cancer cell lines were cultured in Dulbecco's Modified Eagle Medium (DMEM) supplemented with 10% fetal bovine serum (FBS), 1% Pen/Strep (Bio-Concept) and 1% MEM-NEAA (MEM non-essential amino acids, ThermoFisher Scientific). NCM460D normal colonic cell lines[11,12] were purchased from INCELL Corporation and grown in M3 Base Media (serum-free), according to instructions. All cell lines were authenticated by short tandem repeats as previously described [13]. Cultures were confirmed to be free of mycoplasma infection using the PCR-based Universal Mycoplasma Detection kit, as previously described [14].

Transient gene knockdown and overexpression

For transient gene knockdown, log-phase SW480 and DLD-1 colorectal cancer cells were seeded at approximately 60% confluence in 6-well plates and transfected with siRNA against

human ADSL (Dharmacon, #L-010986-00-0005) or non-targeting control siRNA (Dharmacon, #D-001810-10-20) to a final concentration of 25 nM, according to the manufacturer's protocol. Cells were harvested at 24, 48 and 72 h post-transfection for protein isolation. For gene overexpression, log-phase Caco-2 and HT-29 colorectal cancer cells were seeded in 6-well plates at approximately 60%-80% confluence and transfected with pLV-EGFP:T2A:Puro-CMV>Luc2 (Vectorbuilder, #VB190320-1059xxv) or pLV-EGFP:T2A:Puro-CMV>hADSL(NM_000026.4) (Vectorbuilder, #VB190913-1149njg) expression vectors. For both gene knockdown and overexpression assays 8 hours after transfection, the antibiotic-free medium was replaced with a complete medium.

Immunoblot

Total protein extract were obtained by directly lysing cells in Co-IP buffer (100 mmol/L NaCl, 50 mmol/L Tris pH 7.5, 1 mmol/L EDTA, 0.1% Triton X-100) containing 1x protease inhibitors (cOmplete Mini, EDTA-free Protease Inhibitor Cocktail #4693159001, Roche) and 1x phosphatase inhibitors (PhosSTOP #4906837001, Merck). Cell lysates were then treated with 10x reducing agent (NuPAGE Sample Reducing Agent #NP0009, Invitrogen), 4x loading buffer (NuPAGE LDS Sample Buffer #NP0007, Invitrogen), boiled and loaded into neutral pH, pre-cast, discontinuous SDS-PAGE mini-gel system (NuPAGE 4-12% Bis-Tris Protein Gels, #NP0322BOX, Invitrogen). Proteins were then transferred to nitrocellulose membranes using the Trans-Blot Turbo Transfer System (Bio-Rad). Membranes were blocked in 3% Sure Block (Lubio Science, #SB232010-250G) for 1 hour and then incubated overnight at 4˚C with the primary antibodies. Secondary goat anti-mouse (IRDye 680) or anti-rabbit (IRDye 800) antibodies were both from LI-COR Biosciences. Blots were scanned using the Odyssey Infrared Imaging System (LI-COR Biosciences).

Cell proliferation assay

24 hours after transfection 5000 cells were seeded in a 96-well plate. After 4, 24, 48, 72 and 96 hours, CellTiter-Glo^{TM} reagent was added directly into each well and incubated 10 minutes

at room temperature. Luminescence signal was measured using Varioskan Microplate Reader (Thermofisher Scientific). Data were normalized to the 4 hours value. For the rescue experiment cells were seeded 24 and 48 h after the first and the second transfection, respectively.

Migration assay

Briefly, 24 hours after transfection, cells were seeded in CIM-plate 16 (ACEA Biosciences) and FBS was used as a chemoattractant, following manufacturer's instructions. CIM-plates were loaded in the RTCA machine and cell impedance (cell index) was measured continuously every 15 minutes for 36 hours (corresponding to 60 hours after transfection). Briefly, wells of the lower chamber were filled with 170 µL of culture medium with 10% FBS. The upper chamber was then placed onto the lower chamber and 50 µL of serum free medium was added on each CIM well for the background measurement. Next, 7-10x10^4 cells resuspended in 100 µl medium with 1% FBS were added to each well of the upper chamber.

Cell cycle analysis by flow cytometry

Briefly, cells were harvested, washed twice in PBS at 1.200 rpm for 5 minutes, and fixed in 70% Et-OH overnight at 4°C. Cells were then washed twice in PBS at 2000-2200 rpm for 10 minutes and afterward resuspended in 0.5-2.0 mL of DAPI stain solution (0.1% TritonX 100 and 10 µg/mL). After 30 minutes of incubation on ice, cells were analyzed by flow cytometry, measuring the fluorescence emission at 461 nm.

Generation of CRC patient-derived organoids

For the generation of patient-derived organoids tissue was cut into small pieces and subsequently digested in 5 mL advanced DMEM/F-12 (GIBCO, #12634028) containing 2.5 mg/mL collagenase IV (Worthington, #LS004189), 0.1 mg/mL DNase IV (Sigma, #D5025), 20 ug/mL hyaluronidase V (Sigma, #H6254), 1% BSA (Sigma, #A3059) and 10 µM LY27632 (Abmole Bioscience, #M1817) for 1 hour and 30 minutes at 37°C under slow rotation and

vigorous pipetting every 15 minutes. The tissue lysate was filtered through a 100 µM cell strainer, centrifuged at 300 g for 10 minutes and then treated with Accutase (Sigma, #A6964) for 15 minutes at room temperature in order to dissociate the remaining fragments. After 5 minutes centrifugation at 300 g, the cell pellet was finally suspended with growth factor reduced Matrigel (Corning, #356231) and seeded as drops in a tissue-culture dish. After polymerization of Matrigel, medium supplemented with growth factors was added to the cells. Medium was changed every 3 days and organoids were passaged after dissociation with 0.25% Trypsin-EDTA (GIBCO, #25200056).

Chorioallantoic membrane (CAM) assay

Fertilized chicken eggs were obtained from local hatchery (Gepro Geflügelzucht AG, Flawil). Cells were harvested 24 hours post transfection cells were detached from the culture dish with Trypsin, counted, suspended in 10 µl of medium (DMEM) and mixed with an equal volume of Matrigel (Matrigel® #354234; CORNING). For drug treatment, 24 hours after transfection, cells were treated with 6-MP at a final concentration of 2.5 µM or with equal volume of DMSO and after additional 24 hours harvested for inoculation in the CAMs. After inoculation, the windows on the egg shell were sealed with sterile dressings (Mefix, Mölnlycke).

RNA extraction and quantitative PCR

Tumor tissues were homogenized using a Bioruptor (Diagenode) and a minichiller (Huber) during 10 cycles (30 seconds on and 30 seconds off). cDNA was synthesized using SuperScript™ IV VILO™ Master Mix with ezDNase Enzyme (Invitrogen, #11766050). All reverse transcriptase reactions (RT-PCR) were conducted on an Applied Biosystem 7900HT thermocycler. Quantitative RT-PCR analysis was performed using FastStart Universal SYBR Green Master Mix (Sigma-Aldrich, #4913850001) on a QuantStudio 3 Real-Time PCR System (Applied Biosystems) under the following cycling conditions: 1 cycle 50 °C for 2 minutes, 95 °C for 10 minutes, and then 50 cycles of 95 °C for 15 seconds and 60 °C for 1 minutes. The following primers were used: ADSL 5'-TCT TTC ACT GGT CGT GCC TC -3'; 3`-TCT GCT

TTC ACC TTC ATC ACG-5`; GAPDH 5'-AGG TGA AGG TCG GAG TCA ACG-3'; 3`-TGG AAG ATG GTG ATG GGA TTT-5`.

The specificity of the reaction was verified by melting curve analysis.

Metabolite extraction (sample preparation, protein quantification, sample amount normalization)

Cell lysates were extracted by the addition of MeOH:H2O (4:1) (1.5 mL) [15,16]. This solution containing lysed cells was further homogenized in the Cryolys Precellys 24 sample Homogenizer (2 x 20 seconds at 10000 rpm, Bertin Technologies, Rockville, MD , US) with ceramic beads. The bead beater was air-cooled down at a flow rate of 110 L/min at 6 bar. Homogenized extracts were centrifuged for 15 minutes at 4000 g at 4°C (Hermle, Gosheim, Germany) and the resulting supernatant was collected and evaporated to dryness in a vacuum concentrator (LabConco, Missouri, US).

The protein pellets were evaporated and lysed in 20 mM Tris-HCl (pH 7.5), 4M guanidine hydrochloride, 150 mMNaCl, 1 mM Na2EDTA, 1 mM EGTA, 1% Triton, 2.5 mM sodium pyrophosphate, 1 mM beta-glycerophosphate, 1mM Na3VO4, 1 µg/ml leupeptin using the Cryolys Precellys 24 sample Homogenizer (2 x 20 seconds at 10000 rpm, Bertin Technologies, Rockville, MD , US) with ceramic beads.BCA Protein Assay Kit (Thermo Scientific) was used to measure (A562nm) total protein concentration (Hidex).

Samples were normalized based on the total protein content prior to the LC-MS/MS analysis by resuspending the dried extract in MeOH:H2O (4:1, v/v).

Multiple pathway targeted analysis (Data acquisition – LC-MS Analysis)

Extracted samples were analyzed by Hydrophilic Interaction Liquid Chromatography coupled to tandem mass spectrometry (HILIC - MS/MS) in both positive and negative ionization modes using a 6495 triple quadrupole system (QqQ) interfaced with 1290 UHPLC system (Agilent Technologies) [17].

In positive mode, the chromatographic separation was carried out in an Acquity BEH Amide, 1.7 µm, 100 mm × 2.1 mm I.D. column (Waters). Mobile phase was composed of A = 20 mM ammonium formate and 0.1 % FA in water and B = 0.1 % formic acid in ACN. The linear gradient elution from 95% B (0-1.5 min) down to 45% B was applied (1.5 min -17 min) and these conditions were held for 2 min. Then initial chromatographic conditions were maintained as a post-run during 5 min for column re-equilibration. The flow rate was 400 µL/min, column temperature 25 °C and sample injection volume 2µl. ESI source conditions were set as follows: dry gas temperature 290 °C, nebulizer 35 psi and flow 14 L/min, sheath gas temperature 350 °C and flow 12 L/min, nozzle voltage 0 V, and capillary voltage 2000 V. Dynamic Multiple Reaction Monitoring (DMRM) was used as acquisition mode with a total cycle time of 600 ms. Optimized collision energies for each metabolite were applied.

In negative mode, a SeQuant ZIC-pHILIC (100 mm, 2.1 mm I.D. and 5 µm particle size, Merck) column was used. The mobile phase was composed of A = 20 mM ammonium Acetate and 20 mMNH4OH in water at pH 9.7 and B = 100% ACN. The linear gradient elution from 90% (0-1.5 min) to 50% B (8-11min) down to 45% B (12-15 min). Finally, the initial chromatographic conditions were established as a post-run during 9 min for column re-equilibration. The flow rate was 300 µL/min, column temperature 30 °C and sample injection volume 2µl. ESI source conditions were set as follows: dry gas temperature 290 °C and flow 14L/min, sheath gas temperature 350 °C, nebulizer 45 psi, and flow 12 L/min, nozzle voltage 0 V, and capillary voltage -2000 V. Dynamic Multiple Reaction Monitoring (dMRM) was used as acquisition mode with a total cycle time of 600 ms. Optimized collision energies for each metabolite were applied.

Pooled QC samples (representative of the entire sample set) were analyzed periodically (every 7 samples) throughout the overall analytical run in order to assess the quality of the data, correct the signal intensity drift *(if any, this drift is inherent to LC-MS technique and MS detector due to sample interaction with the instrument over time*) and remove the peaks with poor reproducibility (CV > 25%) [18]. In addition, a series of diluted quality controls (dQC) were prepared by dilution with methanol: 100% QC, 50%QC, 25%QC, 12.5%QC and 6.25%QC and analyzed at the beginning and at the end of the sample batch. This QC dilution series served as a linearity filter to remove the features which don't respond linearly or correlation with dilution factor is < 0.65 [19].

Metabolomics data pre-processing and analysis

Raw LC-MS/MS data was processed using the Agilent Quantitative analysis software (version B.07.00, MassHunterAgilent technologies). Relative quantification of metabolites was based on EIC (Extracted Ion Chromatogram) areas for the monitored MRM transitions. The obtained tables (containing peak areas of detected metabolites across all samples) were exported to "R" software http://cran.r-project.org/ signal intensity drift correction was done within the LOWESS/Spline normalization program[20] followed by filtering of "not-well behaving" peaks (CV(QC peaks) > 25% & R[15] (QC dilution curve) < 0.65).

RNA sequencing and pathway analysis

RNA samples were treated with Turbo DNase (AM 1907, Thermo Fisher Scientific) and quantified using a Qubit Fluorometer (Life Technologies). RNA integrity was measured using the Agilent Bioanalyzer 2100 (Agilent Technologies). Library generation was performed using the TruSeq Stranded mRNA protocol (Illumina). Paired-end 150 bp sequencing protocol was performed on the Illumina NovaSeq 6000 platform according to the manufacturer's guidelines. Reads were aligned to the GRCh37 human reference genome using STAR 2.7.1 [21], and transcript quantification was performed using RSEM 1.3.2 [22]. Genes without at least ten

assigned reads in at least two samples were discarded. Counts were normalized using the median of ratios method from the DESeq2 package [23] in R version 3.6.1 (https://www.R-project.org/).

Supplementary References

1. Mesker WE, Junggeburt JMC, Szuhai K, et al. The carcinoma-stromal ratio of colon carcinoma is an independent factor for survival compared to lymph node status and tumor stage. Cell Oncol. 2007; 29: 387–98.

2. Kishore S, Piscuoglio S, Kovac MB, Gylling A, Wenzel F. 3′-UTR poly (T/U) tract deletions and altered expression of EWSR1 are a hallmark of mismatch repair–deficient cancers. Cancer Res. 2014; 74: 224-34.

3. Cancer Genome Atlas Network. Comprehensive molecular characterization of human colon and rectal cancer. Nature. 2012; 487: 330–7.

4. Colaprico A, Silva TC, Olsen C, et al. TCGAbiolinks: An R/Bioconductor package for integrative analysis of TCGA data. Nucleic Acids Res. 2016; 44: e71.

5. Uhlen M, Zhang C, Lee S, et al. A pathology atlas of the human cancer transcriptome. Science. 2017; 19: 786-795.

6. Robinson MD, McCarthy DJ, Smyth GK. edgeR: a Bioconductor package for differential expression analysis of digital gene expression data. Bioinformatics. 2010; 26: 139–40.

7. Robinson MD, Oshlack A. A scaling normalization method for differential expression analysis of RNA-seq data. Genome Biol. 2010; 11: R25.

8. [Internet] Sergushichev AA. An algorithm for fast preranked gene set enrichment analysis using cumulative statistic calculation. 2016. https://github.com/ctlab/fgsea

9. Subramanian A, Tamayo P, Mootha VK, et al. Gene set enrichment analysis: a knowledge-based approach for interpreting genome-wide expression profiles. Proc Natl Acad Sci U S A. 2005; 102: 15545–50.

10. Eide PW, Bruun J, Lothe RA, Sveen A. CMScaller: an R package for consensus molecular subtyping of colorectal cancer pre-clinical models. Sci Rep. 2017; 7: 16618.

11. Li H, Rong S, Chen C, et al. Disparate roles of CXCR3A and CXCR3B in regulating progressive properties of colorectal cancer cells. Mol Carcinog. 2019; 58: 171–84.

12. Ma C-T, Luo H-S, Gao F, Tang Q-C, Chen W. promotes the progression of colorectal cancer by interacting with E-cadherin. Oncol Lett. 2018; 16: 2606–12.

13. Parson W, Kirchebner R, Mühlmann R, et al. Cancer cell line identification by short tandem repeat profiling: power and limitations. FASEB. 2005; 19:1-18.

14. Ng CKY, Martelotto LG, Gauthier A, et al. Intra-tumor genetic heterogeneity and alternative driver genetic alterations in breast cancers with heterogeneous HER2 gene amplification. Genome Biol. 2015; 16: 107.

15. Roci I, Gallart-Ayala H, Schmidt A, et al. Metabolite Profiling and Stable Isotope Tracing in Sorted Subpopulations of Mammalian Cells. Anal Chem. 2016; 88: 2707-13.

16. Ivanisevic J, Zhu Z-J, Plate L, et al. Toward 'Omic Scale Metabolite Profiling: A Dual Separation–Mass Spectrometry Approach for Coverage of Lipid and Central Carbon Metabolism. Anal Chem. 2013; 85: 6876-84.

17. Gallart-Ayala H, Konz I, Mehl F, et al. A global HILIC-MS approach to measure polar human cerebrospinal fluid metabolome: Exploring gender-associated variation in a cohort of elderly cognitively healthy subjects. Anal Chim Acta. 2018; 1037: 327-337.

18. Dunn WB, The Human Serum Metabolome (HUSERMET) Consortium, Broadhurst D, et al. Procedures for large-scale metabolic profiling of serum and plasma using gas chromatography and liquid chromatography coupled to mass spectrometry. Nature Protocols. 2011; 6: 1060-83.

19. Gagnebin Y, Tonoli D, Lescuyer P, et al. Metabolomic analysis of urine samples by UHPLC-QTOF-MS: Impact of normalization strategies. Anal Chim Acta. 2017; 955: 27–35.

20. Tsugawa H, Kanazawa M, Ogiwara A, Arita M. MRMPROBS suite for metabolomics using large-scale MRM assays. Bioinformatics. 2014; 30: 2379-80.

21. Dobin A, Davis CA, Schlesinger F, et al. STAR: ultrafast universal RNA-seq aligner. Bioinformatics. 2013; 29: 15–21.

22. Li B, Dewey CN. RSEM: accurate transcript quantification from RNA-Seq data with or without a reference genome. BMC Bioinformatics. 2011; 12: 323.

23. Love MI, Huber W, Anders S. Moderated estimation of fold change and dispersion for RNA-seq data with DESeq2. Genome Biol. 2014; 15: 550.

Supplementary Tables, Supplementary Figures and Supplementary Legends

Clinical Features	Frequency		p-value
	Low ADSL expression	High ADSL expression	
**Age**			0.127
<59	**0 (0%)**	**7 (100%)**	
60-69	**7 (33%)**	**14 (66%)**	
70-79	**10 (48%)**	**11 (52%)**	
>80	**11 (44%)**	**14 (56%)**	
**Sex**			1
Male	17 (39%)	27 (61%)	
Female	11 (37%)	19 (63%)	
**Tumor Location**			**0.029**
Caecum	4 (31%)	9 (69%)	
Ascending Colon	**10 (59%)**	**7 (41%)**	
Transverse Colon	0 (0%)	2 (100%)	
Descending Colon	5 (83%)	1 (17%)	
Sigmoid Colon	5 (25%)	15 (75%)	
Rectum	4 (25%)	12 (75%)	
**Stage**			**0.026**
I	**3 (20%)**	**12 (80%)**	
II	**5 (28%)**	**13 (72%)**	
III	**8 (35%)**	**15 (65%)**	
IV	**12 (67%)**	**6 (33%)**	
**Grade**			0.054
Low	**17 (31%)**	**38 (69%)**	
High	**11 (58%)**	**8 (42%)**	
**Tumor:Stroma**			**0.001**
Stroma Low	17 (29%)	42 (71%)	
Stroma High	11 (79%)	3 (21%)	
**Microsatellite Instability**			0.242
MSI	1 (14%)	6 (86%)	
MSS	27 (40%)	40 (60%)	
**Lymphatic invasion**			**0.001**
Negative	**9 (23%)**	**31 (78%)**	
Positive	**15 (65%)**	**8 (35%)**	
**Venous Invasion**			**0.016**
Negative	**14 (30%)**	**33 (70%)**	
Positive	**10 (67%)**	**5 (33%)**	
**Lymphovascular Invasion**			**0.001**

Negative	8 (22%)	28 (78%)	
Positive	19 (63%)	11 (37%)	

All 2x2 contingency tables were analysed with fisher's exact tests. All others by chi-squared test

Table S1: Association between ADSL (protein and mRNA) expression and clinicopathologic features in human samples and clinicopathologic information of the patient derived organoids. Statistical comparisons were performed using Fisher’s exact test, Chi-squared test and Cochran-Armitage test.

Clinical and Molecular Features	Low ADSL expression n (%)	High ADSL expression n (%)	P-value	
**Sex (n=591)**	Female (n=271)	181 (66.8%)	90 (33.2%)	0.6
	Male (n=320)	207 (64.7%)	113 (35.3%)	
**AJCC Stages (n=571)****	Stage I + II (n=316)	193 (61.1%)	123 (38.9%)	**0.03**
	Stage III + IV (n=255)	178 (69.8%)	77 (30.2%)	
**CRC Subtyping (n=556)****	CMS1 (n=97)	67 (69.1%)	30 (30.9%)	**<0.001**
	CMS2 (n=170)	85 (50.0%)	85 (50.0%)	
	CMS3 (n=94)	66 (70.2%)	28 (29.8%)	
	CMS4 (n=195)	145 (74.4%)	50 (25.6%)	

** Patients with Not Available, Unknown and Discrepanices

Table S2: Association between *ADSL* mRNA expression and clinicopathological features in the TCGA cohort

Organoid ID	Age	Sex	stage at diagnosis	grade	tumor location	chemo regime prior to resection metastasis	Clinical response
P1	69	male	IV (T3 N0 L0 V0 Pn0 R0 M1)	G2	colon sigmoideum	3 cycles Oxaliplatin+ 5-FU	TRG 0, progression in size
P2	81	female	IIa (T3 N0 L0 V0 Pn0 R0 M0)	G2	coecum	none	Ø
P3	61	female	IIa (T3 N0 L0 V0 Pn0 R0 M0)	G2	colon sigmoideum	6 cycles Oxaliplatin+5-FU+Irinotecan+Cetuximab	TRG 2, regression in size
P4	79	female	IV (T4 N0 L0 V0 Pn0 R0 M1)	G3	colon ascendens	3 cycles Oxaliplatin+5-FU	TRG 3, regression in size

Table S3: Clinicopathological features patients deriving organoids

Figure S1

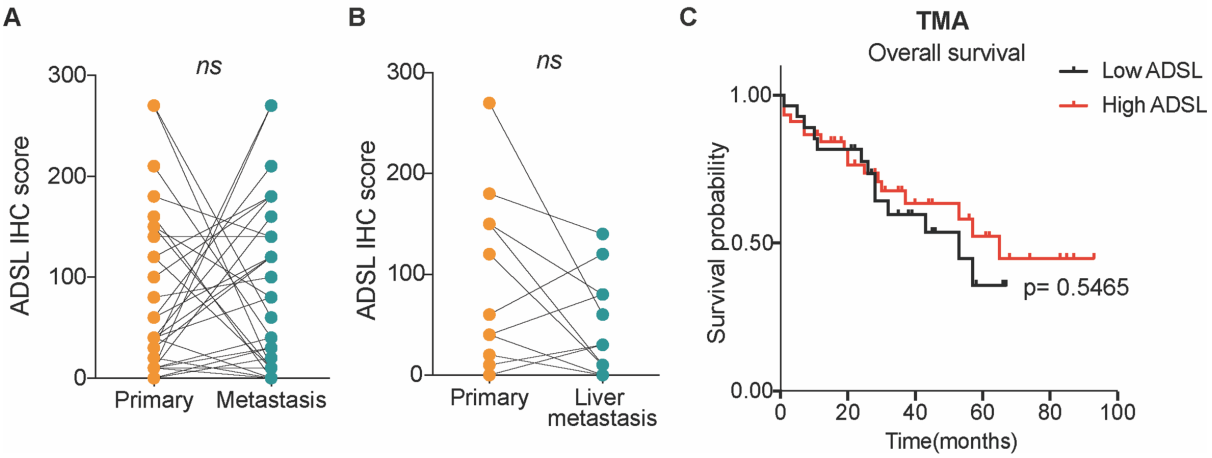

Figure S1: (**A**) Semi-quantitative scoring of ADSL staining in CRC primary tumors and paired metastases. (**B**) Semi-quantitative scoring of ADSL staining in CRC primary tumors and paired liver metastases. (**C**) Kaplan-Meier curves show the overall survival for patients with CRC primary tumors stratified by high and low ADSL staining. Statistical significance for (**A,B**) was assessed by paired student's t-test and for (**C**) by log-rank test.

Figure S2

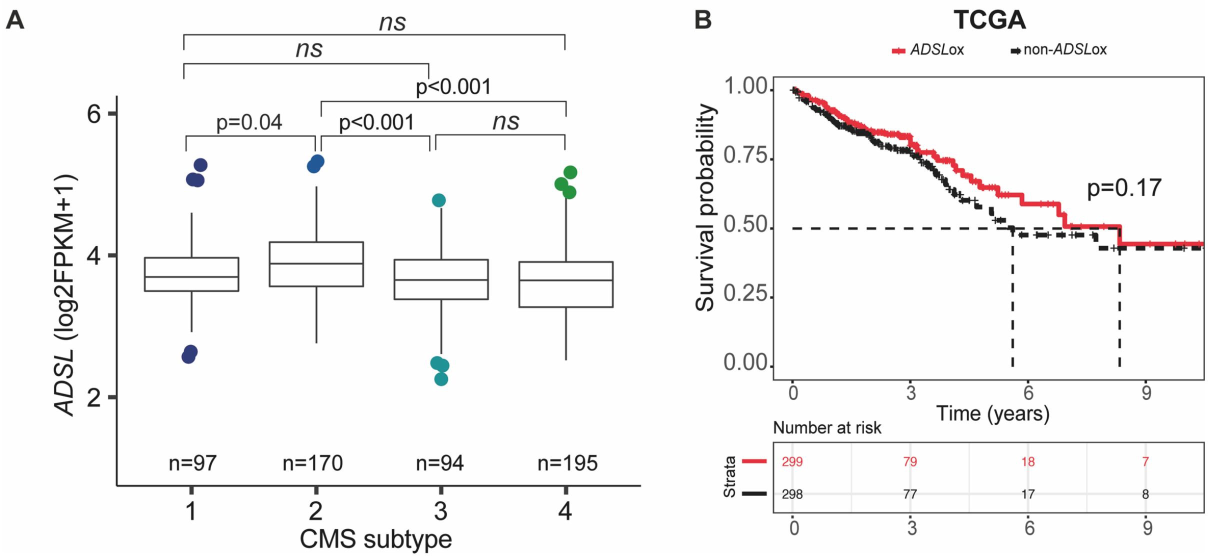

Figure S2: (**A**) *ADSL* transcript expression in CRCs stratified by the consensus molecular subtypes (CMS). (**B**) Kaplan-Meier curves show the overall survival for 622 CRC patients from the TCGA cohort [37], stratified by high and low *ADSL* RNA expression levels. Statistical comparison was performed by the log-rank test. Statistical significance for (**B**) was assessed by unpaired student's t-tests.

Figure S3

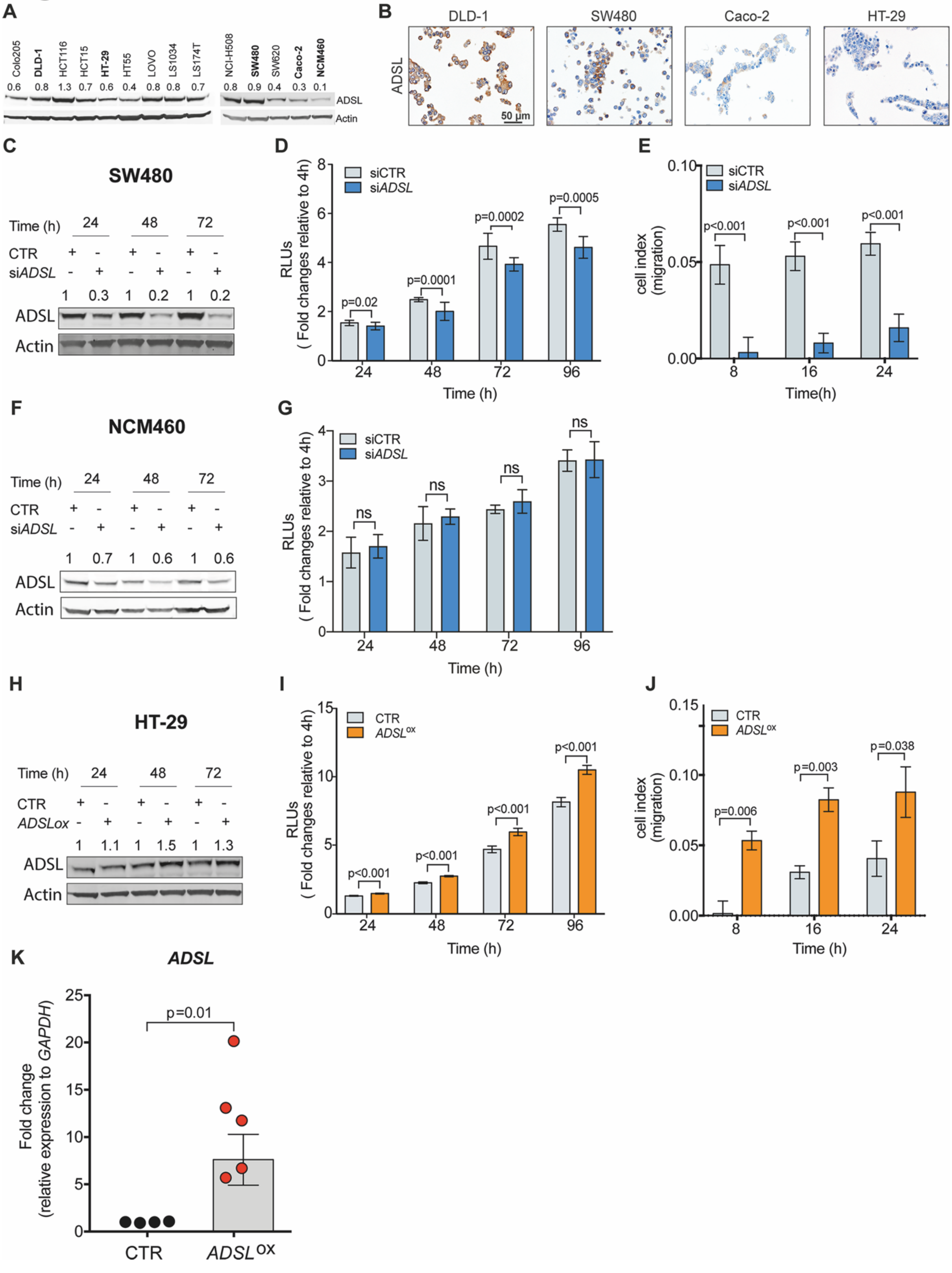

Figure S3: (**A**) Immunoblot showing endogenous ADSL expression in a panel of CRC cell lines and normal colonic cells NCM460. (**B**) Representative micrographs of DLD-1, SW480,

Caco-2 and HT-29 colorectal cells immunostained with ADSL. Scale bar 50 μm. (**C**) Immunoblot showing ADSL expression in SW480 cells at 24, 48 and 72 h post-transfection with siRNA against *ADSL* or siRNA control. (**D**) Proliferation and (**E**) migration capacity of SW480 *ADSL*-silenced cells compared to control cells. (**F**) Immunoblot showing *ADSL* expression in NCM460 cells at 24, 48 and 72 h post-transfection with siRNA against *ADSL* or siRNA control. (**G**) Proliferation capacity of NCM460 *ADSL*-silenced cells compared to control cells. (**H**) Immunoblot showing *ADSL* expression in HT-29 control or *ADSL*-overexpressed cells at 24, 48 and 72 h post transfection. (**I**) Proliferation and (**J**) migration capacity of HT-29 *ADSL*-overexpressing cells compared to control cells. For all immunoblots quantification is relative to the loading control (actin). (**K**) *ADSL* mRNA levels of CAM-engrafted tumors derived from Caco-2 control or *ADSL*-overexpressed cells. For all experiments, statistical significance was assessed by multiple t-tests. Error bars represent mean +/- SD.

Figure S4

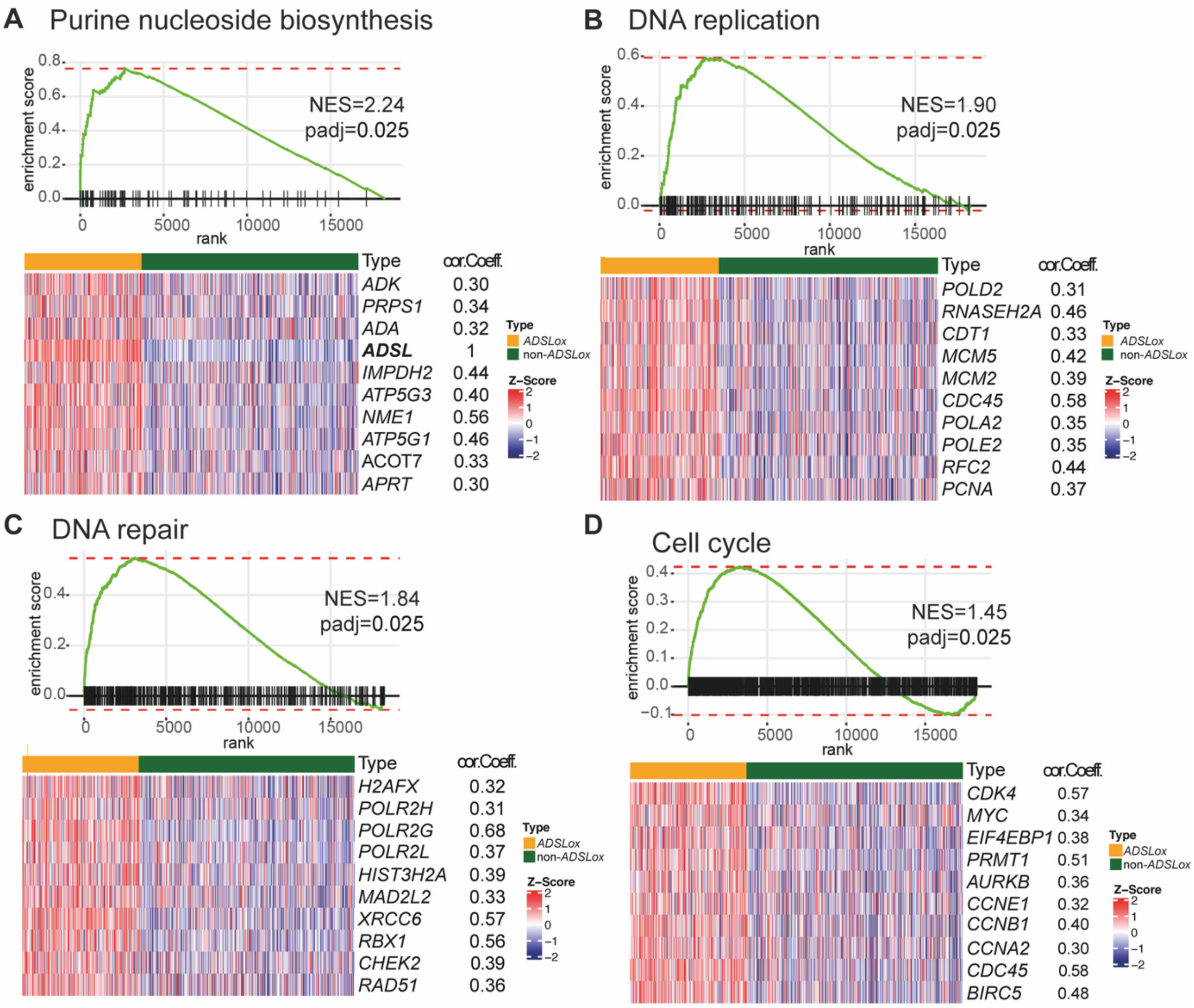

Figure S4: Gene Set Enrichment Analysis plots of Gene Ontology (GO) (**A**) Purine nucleoside biosynthetic process, (**B**) DNA replication, (**C**) DNA repair, (**D**) cell cycle, where x-axis shows ranked list of genes (ranked by the p-values signed according to the direction of the differential expression analysis between *ADSL*ox and non-*ADSL*ox CRCs) and the vertical bars along the x-axis show the genes that belong to gene set. The y-axis shows the enrichment score of the gene set. Heatmaps below show selected genes in the GO processes. NES=normalized enrichment score.

Figure S5

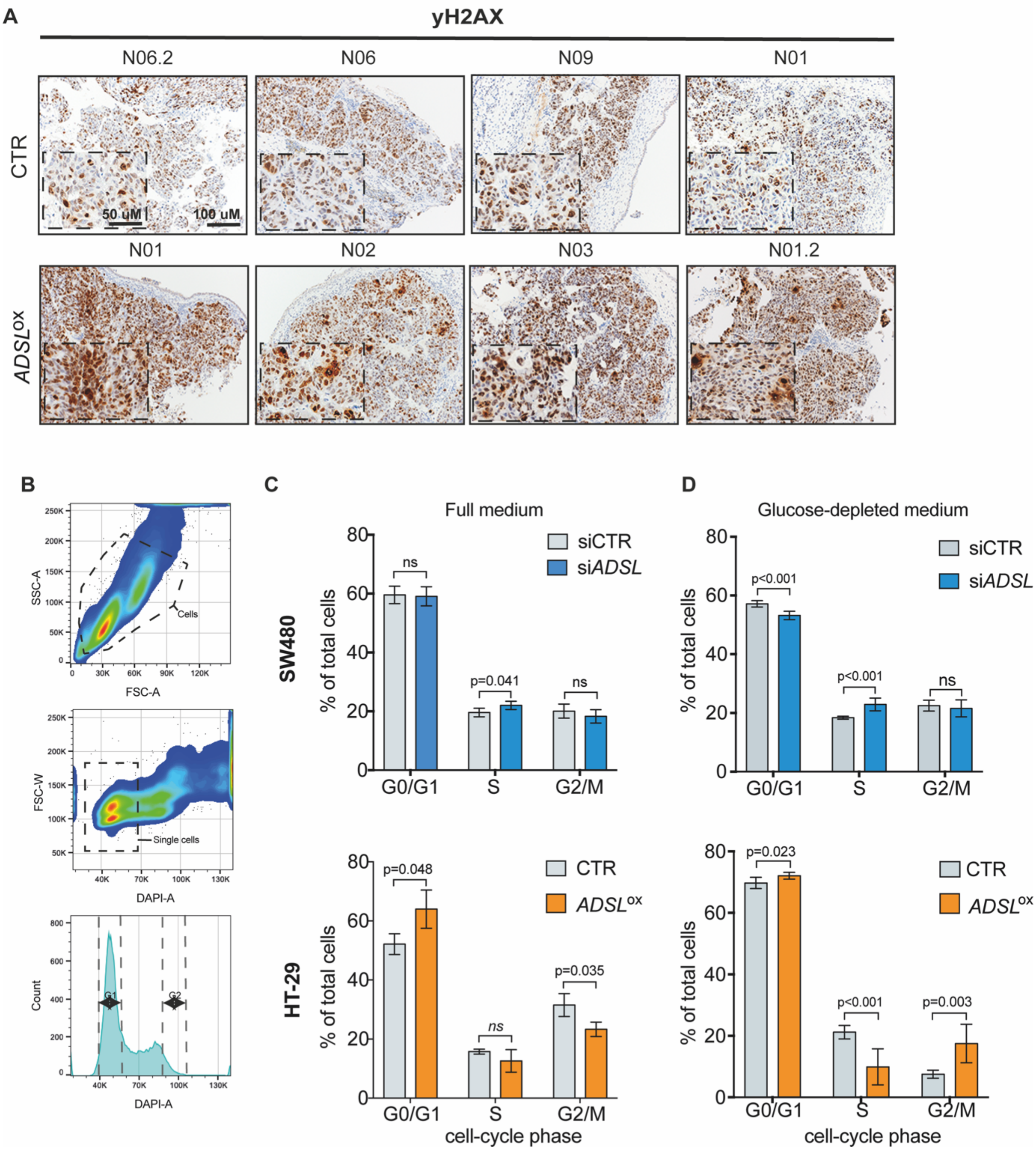

Figure S5: (**A**) Representative pictures of Caco-2 tumors extracted 4 days post-implantation. Tissue sections were immunostained with the DNA damage marker γH2AX. Scale bars 50-100 μm. (**B**) Flow cytometry gating strategy used for cell cycle analysis. (**C-D**) FACS analysis of DAPI-stained CRC cell lines (SW480 in blue; HT-29 in orange) upon *ADSL* transient downregulation or upregulation in (**C**) full medium and (**D**) glucose-deprived condition. For all

experiments, statistical significance was assessed by multiple t-tests. Error bars represent mean +/- SD.

Figure S6

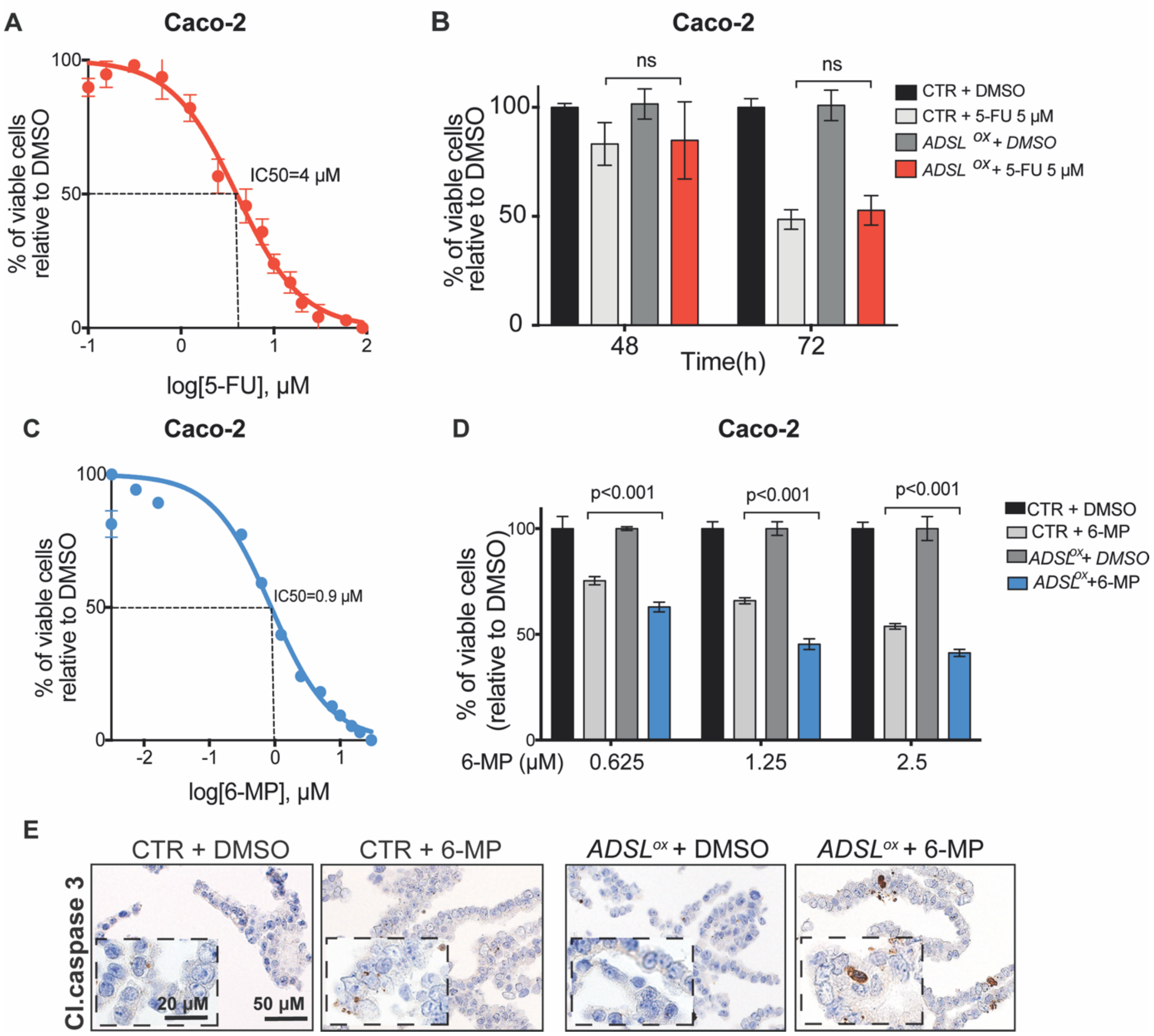

Figure S6: (**A,C**) log-dose response of (**A**) 5-FU and (**C**) 6-MP in Caco-2 cells. (**B**) Percentage of cell viability of Caco-2 upon *ADSL* overexpression and/or treatment with 5 μM of 5-FU for 48 and 72 h. (**D**) Percentage of cell viability of Caco-2 upon *ADSL* overexpression and/or treatment with 0.625, 1.25 and 2.5 μM of 6-MP for 48 h. (**E**) Representative pictures of control and *ADSL* overexpression Caco-2 cells immunostained with cleaved caspase 3. Scale bars 20-50 μm. All experiments were performed in duplicate. Error bars represent mean +/- SD. For all experiments, statistical significance was assessed by multiple t-tests.

Figure S7

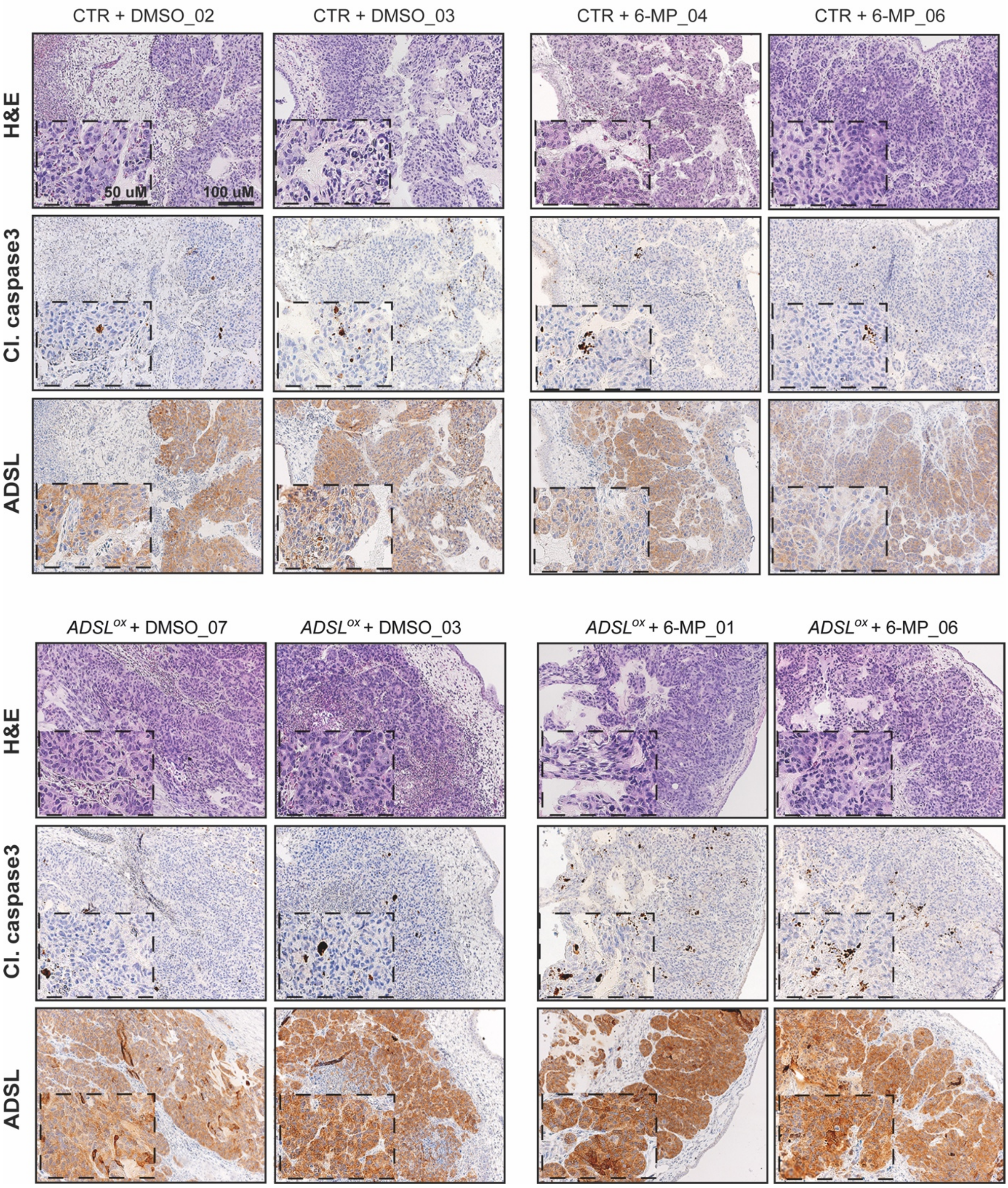

Figure S7: Representative pictures of Caco-2 tumors extracted from CAM 4 days post-implantation. Tissue sections were stained with H&E and treatment conditions. Scale bars 50-100 µm.

Figure S8

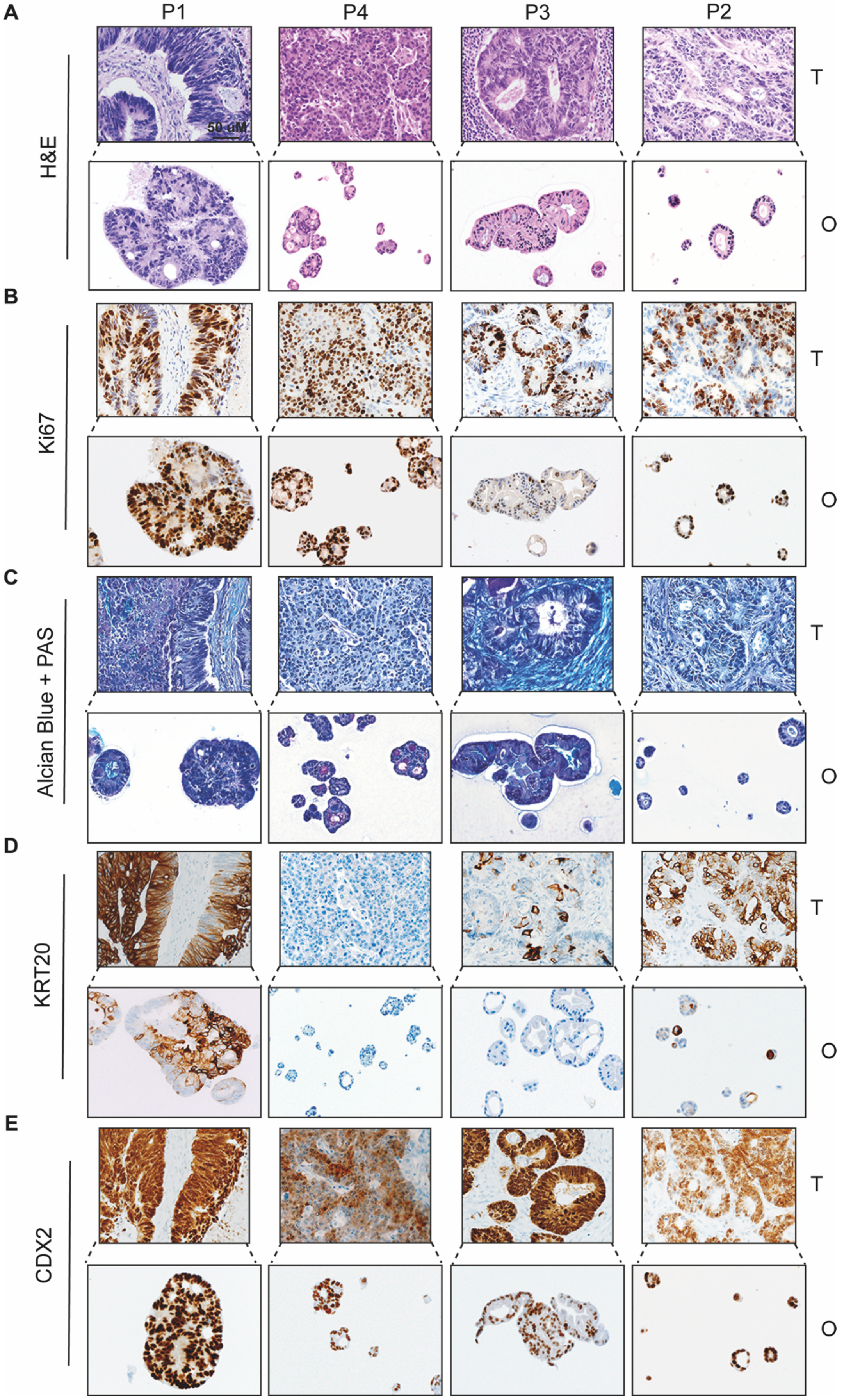

Figure S8: Representative micrographs of (**A**) Hematoxylin and eosin, (**B**) Ki67, (**C**) Alcian blue+PAS, (**D**) Keratin 20 and (**E**) CDX2 of CRC patients liver metastasis (T) and matched derived organoids (O). Scale bar 50 μm.

Figure S9

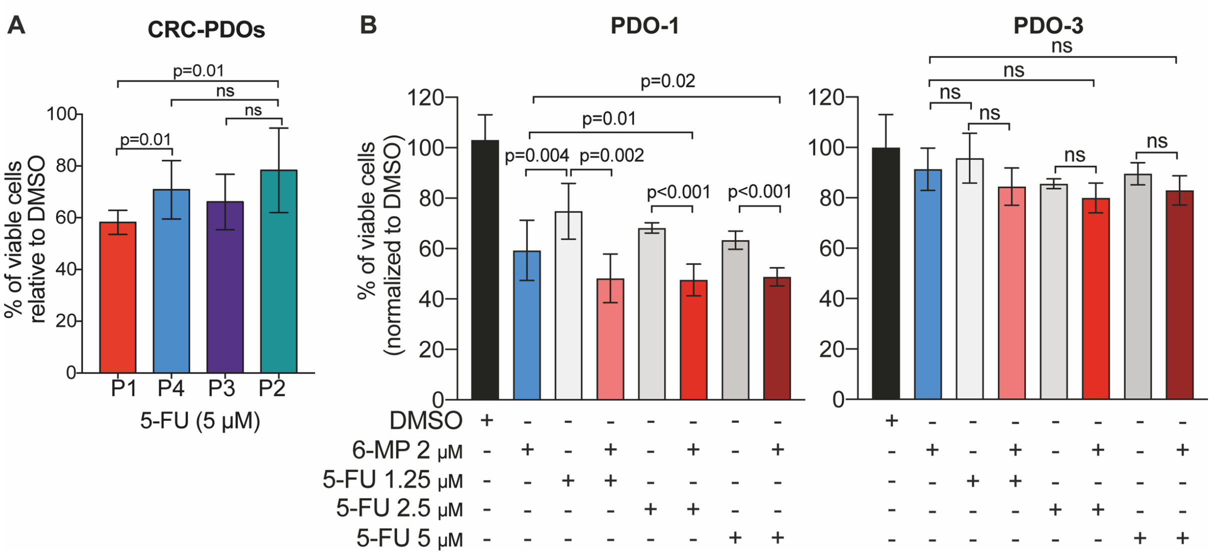

Figure S9. (**A**) Percentage of viability relative to DMSO of CRC-PDOs treated 5 days with 5 µM of 5-FU. (**B**) Percentage of viability relative to DMSO of CRC-PDO1 and CRC-PDO3 treated 5 days with DMSO and 6-MP alone (2.5 µM), 5-FU alone (1.25, 2.5 and 5 µM) or in combination. All experiments were performed in duplicate. Error bars represent mean +/- SD. For all experiments, statistical significance was assessed by multiple t-tests.

Figure S10

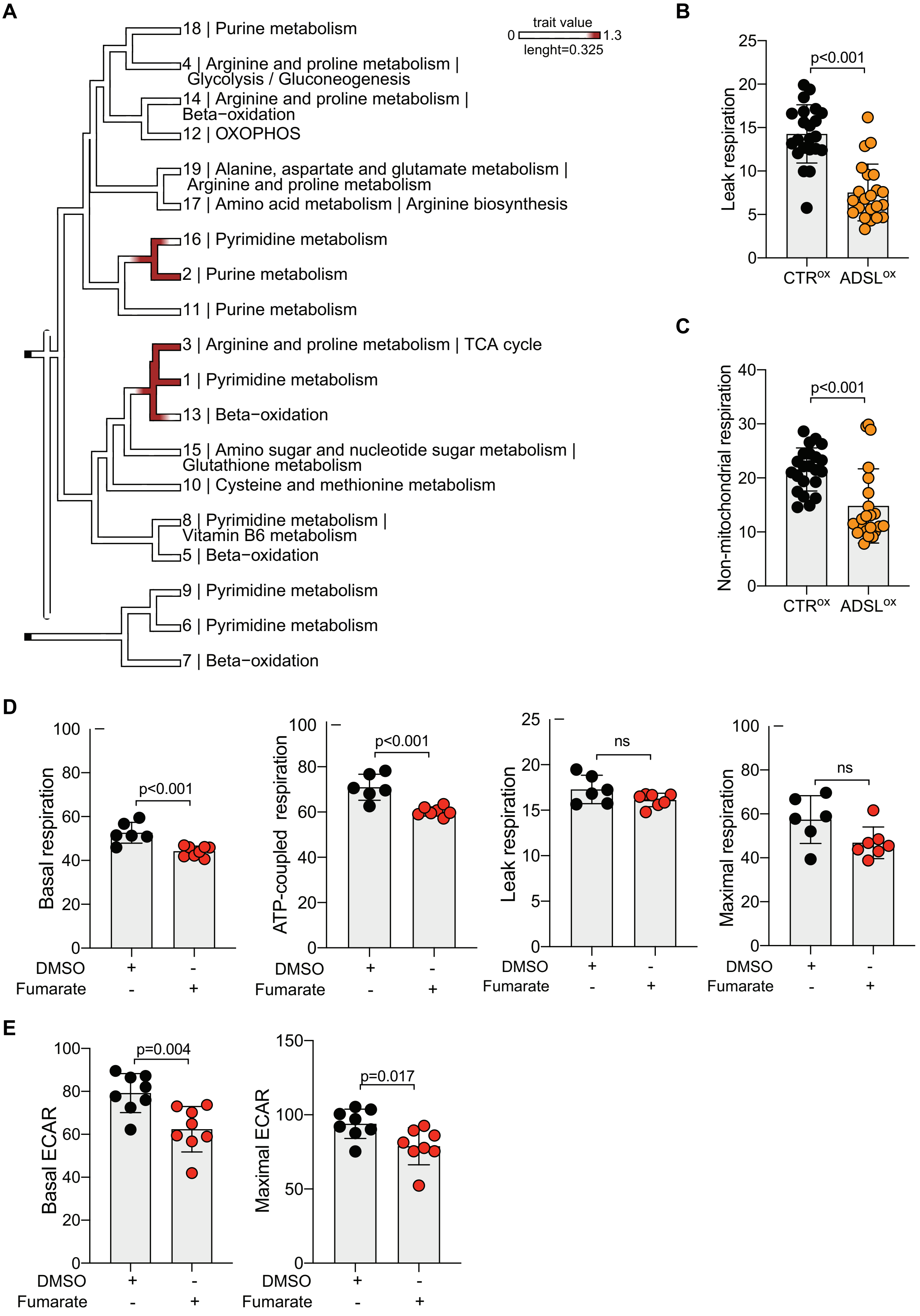
A
trait value
0
1.3
lenght=0.325
18 | Purine metabolism
4 | Arginine and proline metabolism | Glycolysis / Gluconeogenesis
14 | Arginine and proline metabolism | Beta−oxidation
12 | OXOPHOS
19 | Alanine, aspartate and glutamate metabolism | Arginine and proline metabolism
17 | Amino acid metabolism | Arginine biosynthesis
16 | Pyrimidine metabolism
2 | Purine metabolism
11 | Purine metabolism
3 | Arginine and proline metabolism | TCA cycle
1 | Pyrimidine metabolism
13 | Beta−oxidation
15 | Amino sugar and nucleotide sugar metabolism | Glutathione metabolism
10 | Cysteine and methionine metabolism
8 | Pyrimidine metabolism | Vitamin B6 metabolism
5 | Beta−oxidation
9 | Pyrimidine metabolism
6 | Pyrimidine metabolism
7 | Beta−oxidation
B
Leak respiration
p<0.001
CTR^{ox} ADSL^{ox}
C
Non-mitochondrial respiration
p<0.001
CTR^{ox} ADSL^{ox}
D
Basal respiration
p<0.001
ATP-coupled respiration
p<0.001
Leak respiration
ns
Maximal respiration
ns
DMSO + -
Fumarate - +
E
Basal ECAR
p=0.004
Maximal ECAR
p=0.017
DMSO + -
Fumarate - +

Figure S10. (**A**) Association of the ADSL overexpression trait with metabolic correlation modules. Purine metabolism, pyrimidine metabolism, beta-oxidation and TCA cycle modules showed a significant association with higher/lower relative abundance in *ADSL*-overexpressing cells. (**B,C**) Mean leak respiration (B) and non-mitochondrial respiration (C) of control and *ADSL*-overexpressing Caco-2 cells measured after drug-induced mitochondrial stress [75] using Seahorse. (**D**) Mean basal, ATP-coupled, maximal and leak respiration of parental Caco-2 cells treated with DMSO or fumarate (50 μM) measured after drug-induced mitochondrial stress [75] using Seahorse. (**E**) Mean basal and maximal ECAR of the cells in (**D**). All experiments were performed in duplicate. Error bars represent mean +/- SD. For all experiments, statistical significance was assessed by unpaired t-tests.

Figure S11

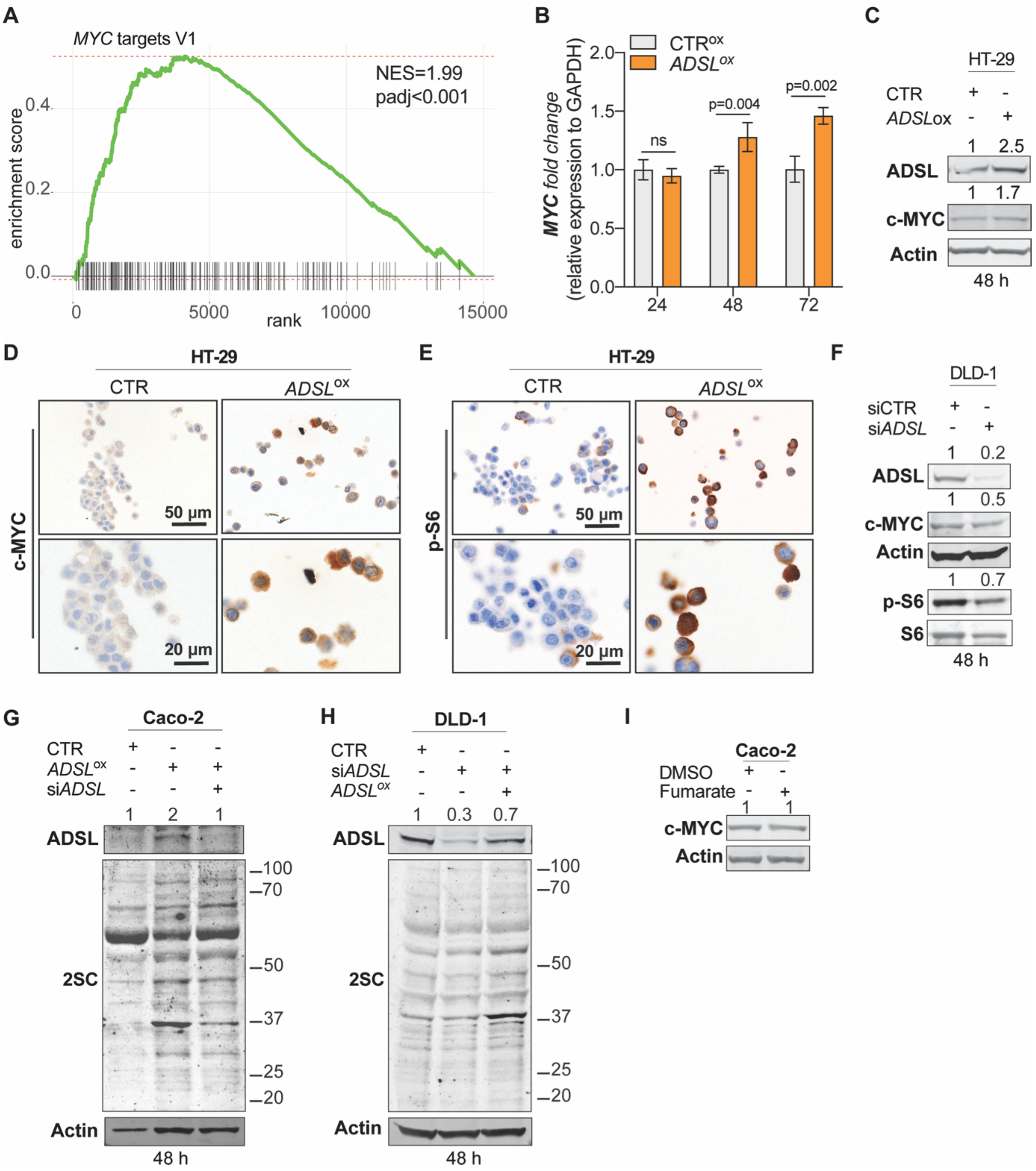

Figure S11. (**A**) Gene Set Enrichment Analysis plots of Hallmark *MYC* targets V1 process. (**B**) *MYC* mRNA expression levels (fold change) in Caco-2 control or *ADSL*-overexpressing cells 24, 48 and 72 h post-transfection. (**C**) Immunoblot of ADSL, c-MYC and Actin in HT-29 control and *ADSL*-overexpressing cells 48 h post transfection. (**D,E**) Representative pictures of HT-29 control and *ADSL*-overexpressing cells immunostained with c-MYC (D) and phospho-S6 (E). Scale bars 50-20 µM. (**F**) Immunoblot of ADSL, c-MYC, total and phospho-

S6 in DLD-1 control and *ADSL*-silenced cells 48 h post-transfection. (**G,H**) Immunoblot of ADSL and succination (2SC) in Caco-2 cells (**G**) or DLD-1 (**H**) 48 h post-transfection. (**I**) Immunoblot of c-MYC in Caco-2 parental cells 24 h post-treatment with fumarate (50 µM).
